# Impaired communication at the neuromotor axis during Degenerative Cervical Myelopathy

**DOI:** 10.3389/fncel.2023.1316432

**Published:** 2024-01-10

**Authors:** Jorge Ojeda, Mayra Vergara, Ariel Ávila, Juan Pablo Henríquez, Michael Fehlings, Pia M. Vidal

**Affiliations:** ^1^Neuroimmunology and Regeneration of the Central Nervous System Unit, Biomedical Science Research Laboratory, Basic Sciences Department, Faculty of Medicine, Universidad Católica de la Santísima Concepción, Concepción, Chile; ^2^Developmental Neurobiology Unit, Biomedical Science Research Laboratory, Basic Sciences Department, Faculty of Medicine, Universidad Católica de la Santísima Concepción, Concepción, Chile; ^3^Neuromuscular Studies Lab (NeSt Lab), Instituto de Anatomía, Histología y Patología, Facultad de Medicina, Universidad Austral de Chile, Valdivia, Chile; ^4^Department of Genetics and Development, Krembil Research Institute, University Health Network, Toronto, ON, Canada; ^5^Spinal Program, University Health Network, Toronto Western Hospital, Toronto, ON, Canada

**Keywords:** Degenerative Cervical Myelopathy, spinal cord, motoneuron, muscle, neuromuscular, synapse

## Abstract

Degenerative Cervical Myelopathy (DCM) is a progressive neurological condition characterized by structural alterations in the cervical spine, resulting in compression of the spinal cord. While clinical manifestations of DCM are well-documented, numerous unanswered questions persist at the molecular and cellular levels. In this study, we sought to investigate the neuromotor axis during DCM. We use a clinically relevant mouse model, where after 3 months of DCM induction, the sensorimotor tests revealed a significant reduction in both locomotor activity and muscle strength compared to the control group. Immunohistochemical analyses showed alterations in the gross anatomy of the cervical spinal cord segment after DCM. These changes were concomitant with the loss of motoneurons and a decrease in the number of excitatory synaptic inputs within the spinal cord. Additionally, the DCM group exhibited a reduction in the endplate surface, which correlated with diminished presynaptic axon endings in the supraspinous muscles. Furthermore, the biceps brachii (BB) muscle exhibited signs of atrophy and impaired regenerative capacity, which inversely correlated with the transversal area of remnants of muscle fibers. Additionally, metabolic assessments in BB muscle indicated an increased proportion of oxidative skeletal muscle fibers. In line with the link between neuromotor disorders and gut alterations, DCM mice displayed smaller mucin granules in the mucosa layer without damage to the epithelial barrier in the colon. Notably, a shift in the abundance of microbiota phylum profiles reveals an elevated Firmicutes-to-Bacteroidetes ratio—a consistent hallmark of dysbiosis that correlates with alterations in gut microbiota-derived metabolites. Additionally, treatment with short-chain fatty acids stimulated the differentiation of the motoneuron-like NSC34 cell line. These findings shed light on the multifaceted nature of DCM, resembling a synaptopathy that disrupts cellular communication within the neuromotor axis while concurrently exerting influence on other systems. Notably, the colon emerges as a focal point, experiencing substantial perturbations in both mucosal barrier integrity and the delicate balance of intestinal microbiota.

## 1 Introduction

Degenerative Cervical Myelopathy (DCM) is an umbrella term that encompasses various compressive myelopathies caused by osteoarthritic changes in the spinal cord. It is a prevalent, debilitating condition affecting mainly older individuals (Irvine et al., 1965; Nurjck, 1972; Young, 2000; Sieck, 2017; Moghaddamjou et al., 2020; Wu et al., 2020). Early narrowing of the spinal canal leads to the sensory and motor hallmark symptoms that include gait and balance abnormalities, leg weakness or stiffness, arm pain, loss of manual dexterity, and abnormal sensations in the hands, as well as gastrointestinal dysfunction (McCormack and Weinstein, 1996; Nouri et al., 2015, 2020). At a different level, chronic compression of the spinal cord triggers microvasculature disruption, reducing the number of endothelial cells, increasing neuronal and oligodendroglial cell apoptosis, and promoting axonal degeneration of the proximal corticospinal segment to the compression site, as well as increasing neuroinflammation (Yu et al., 2011; Karadimas et al., 2015; Jiang et al., 2017; Vidal et al., 2017; Ulndreaj et al., 2022).

According to their primary defects in the spinal cord, DCM patients exhibit marked locomotor phenotypes. This feature is particularly evident in elderly patients, who show marked destruction of the gray matter and a reduced anteroposterior compression ratio of the spinal cord, as well as gait disorders, sensory deficit, muscle wasting, and impaired limb motor function (Ogino et al., 1983; Kalsi-Ryan et al., 2013; Badhiwala et al., 2020; Moghaddamjou et al., 2020). Furthermore, the age-dependent functional decline resulting in energy metabolism imbalance, inflammaging, and muscle weakness, among others, could also contribute to the progression of DCM. In the past, patients with advanced DCM displayed increased spasticity and pain in the limbs, inability to walk, clumsiness of hands, and evident muscle atrophy (Kalsi-Ryan et al., 2013; Akter and Kotter, 2018; Badhiwala et al., 2020; Tetreault et al., 2022). The loss of ventral horn neurons in these patients correlates with both the loss of cellular densities and the formation of small cavities in the gray matter, accompanied by Wallerian degeneration (Ogino et al., 1983; Klironomos et al., 2011; Kubota et al., 2011; Yu et al., 2011; Karadimas et al., 2013; Dhillon et al., 2016). Despite the well-characterized locomotor phenotypes observed in DCM patients, little is known regarding the impact of this myelopathy on peripheral synapses.

The vertebrate neuromuscular junction (NMJ) is the most accessible part of the nervous system that connects the brain stem, spinal cord (SC), and skeletal muscle fibers to voluntary movement. In this synaptic axis, trunks of nerves composed of many single axons go into the muscles to branch into smaller branchlets to finally contact the skeletal muscle endplate. This peripheral cholinergic synapse is constituted by an extra-laminar capping of kranocyte cells, a presynaptic motor nerve terminal, a postsynaptic muscle specialization evidenced by clusters of acetylcholine receptors (AChRs), and non-myelinated terminal Schwann cells (tSCs; Sanes and Lichtman, 2001; Wu et al., 2010; Shi et al., 2012; Tintignac et al., 2015). Multiple pathologies, nerve or muscular injuries, lack of physical activity, and aging impair the synaptic structure and function at the NMJ, resulting in poly-innervation, partial or total denervation, postsynaptic fragmentation, and muscle atrophy, among other phenotypes (Valdez et al., 2010, 2012; Tejero et al., 2016, 2020; Woehlbier et al., 2016; Bermedo-García et al., 2022). However, the behavior of the cellular components of the neuromuscular synapse in the context of DCM has not been studied.

Along with motor alterations in neuromuscular diseases, accumulative evidence has shown the presence of gastrointestinal dysfunction, particularly alterations in the gut microbiota abundance and its derived metabolites (Gungor et al., 2016; Zhang et al., 2018; Lin et al., 2020; Farini et al., 2023). Spinal cord injury (SCI) patients experience neurogenic loss of control over gastrointestinal motility, causing gut dysbiosis (Gungor et al., 2016; Zhang et al., 2018; Lin et al., 2020). Furthermore, growing evidence in amyotrophic lateral sclerosis (ALS), a fatal neurodegenerative disease caused by progressive loss of motoneurons, neuromuscular junction impairment, and muscle atrophy, revealed that gut dysbiosis can exacerbate ALS symptoms (Wu et al., 2015; Zhang et al., 2017; Sun et al., 2019; Di Gioia et al., 2020; Obrenovich et al., 2020; Zeng et al., 2020). This phenotype can be reversed using pro- and prebiotic treatments (Zhang et al., 2017; Blacher et al., 2019; Gotkine et al., 2020). Moreover, the absence of gut microbiota in germ-free mice has been shown to cause skeletal muscle atrophy and decreased expression of genes related to muscle growth and mitochondrial function, a condition that is reverted when germ-free mice are transplanted with gut microbiota from control mice (Lahiri et al., 2019). Additionally, recent studies have highlighted the importance of the gastrointestinal system in DCM pathology. For example, gastrointestinal comorbidities are accounted as one of the vulnerability factors that can influence the development and progression of patients with DCM. Complementing these results, a DCM mouse model has shown changes in the gut microbiota composition during DCM progression. Specifically, there is a reduction of butyrate-producing bacteria and butyrate production (Nouri et al., 2020; Davies et al., 2022; Farkas et al., 2023), suggesting a link between the gut–brain axis and the neuromotor axis.

Even though the understanding of DCM progression has been conditioned by the lack of animal models that accurately reproduce all the features of this pathology (Klironomos et al., 2011; Kalsi-Ryan et al., 2013), here, we have induced DCM in mice by inserting an aromatic polyether material underneath C5-6 laminae to emulate chronic compression of the cervical spinal cord. This model has proven clinical benefits as it robustly mimics in mice the progressive osteoid formation observed in patients (Karadimas et al., 2013; Vidal et al., 2017). After 12 weeks of polymer implantation, the locomotor performance was evaluated using sensorimotor tests. In addition, the pathobiology was analyzed by immunohistochemistry of motoneurons from the cervical SC region, the supraspinatus (SS), and the biceps brachii (BB) skeletal muscles. Our results revealed decreased locomotor activity and strength in the DCM mouse model in comparison with the control group. Remarkably, immunohistochemical co-localization studies showed modified gross anatomy at the cervical SC level, caused by the loss of motoneurons and reducing the number of excitatory synaptic inputs in the DCM group. Furthermore, at the neuromuscular axis, the DCM group exhibited a smaller endplate surface correlating with the area of the presynaptic axon ending in supraspinatus muscles. In addition, the biceps brachii displayed an increased average of cross-sectional area and type I muscle fibers, with an abnormal proportion of centralized nuclei in the DCM group compared to the control group. Furthermore, DCM animals showed smaller mucin granules at the colon and a subsequent dysbiosis, evidenced by an increased Firmicutes rate that could influence the gut microbiota metabolites. Accordingly, treatments with short-chain fat acids butyrate and propionate increased the neurite length in the motoneuron-like NSC34 cell line. Thus, our findings suggest that DCM is a synaptopathy that impairs the structural communication between motoneurons and skeletal muscle fibers required for the correct functional locomotor performance of the upper and lower extremities and could have an influence on the gut microbiota and its derived metabolites.

## 2 Materials and methods

### 2.1 Animal experiments and DCM induction

The experimental procedures were conducted following international standards and the advice of the institutional Committees in place at the *Universidad Católica de la Santísima Concepción,* the current national regulations and standards governing scientific activity in the biological sciences area, and according to the guidelines of the European Council Directive for the Care of Laboratory Animals.

This study was conducted in 8-weeks-old male C57BL/6 mice. We have focused our study on this sex because the incidence of DCM is greater in men versus women and because the male DCM mouse model has shown the highest dysbiosis compared with the female DCM mouse model (Nouri et al., 2022; Farkas et al., 2023). The DCM mouse model was generated using an aromatic polyether polymer as previously described (Karadimas et al., 2013; Ulndreaj et al., 2022). In brief, the aromatic polyether acts as a scaffold for inorganic salts that progressively precipitate, increasing the stenosis of the central canal on the cervical spinal cord segment. Experimental implantation of a small piece of aromatic polyether was conducted under sedation with 2.0% isofluorane at 0.8–1.0 L/min oxygen mixture. Animals were divided into two groups: DCM (*n* = 4) and sham (*n* = 3). A sham was included for comparison, in which the polyether polymer was inserted for 30 s and then removed to simulate and control for any potential minor trauma associated with material insertion (Karadimas et al., 2013; Vidal et al., 2017; Ulndreaj et al., 2022). All mice were examined 24 h after surgery for signs of neurological deficits (Vidal et al., 2017), which resulted in the exclusion of the animal from the study if positive signs were observed (i.e., reduced ankle movement and plantar stepping and upper and/or lower limb stiffness and/or weakness). An age-matched WT group was included as a control during locomotor tests (*n* = 4). After 12 weeks of DCM induction, animals were euthanized by an overdose of isofluorane for histological characterization of SC and selected muscles (Karadimas et al., 2013; Vidal et al., 2017; Ojeda et al., 2020).

### 2.2 Behavioral and locomotor tests

#### 2.2.1 Open-field test

The Standard Operating Procedure for Duchenne Muscular Dystrophy SOP DMD_M.2.1.002 (last reviewed on 12 July 2017) was used with minor modifications to determine general locomotor activity. In brief, mice were placed in the arena for 10 min before recording, allowing free movement through the arena (acclimation; Tatem et al., 2014). The recording was performed for 5 min in a box of 30 cm x 40 cm, and the footage tracking was analyzed at 5 fps using Imaris software. Data collection was used to determine total distance (mm), maximum speed (mm/s), mean speed (mm/s), stopped time, time in the edge, time in the corner, and time in the center of the arena. All of the last ones are expressed as a percentage of the total time recorded.

#### 2.2.2 Pole test

In this study, animals were trained in at least two sessions on consecutive days to perform the tests bi-weekly for 8 weeks after the first month of surgery. Mice were placed on the top of a 50-cm vertical pole with diameters of 6, 8, and 10 mm to discard a cognitive condition. Recording time started when the animal began the turning down movement. The total time analyzed involves the time to turn completely downward and the time to descend until both limbs reach the floor. Measured data are expressed in seconds (Matsuura et al., 1997; Glajch et al., 2012).

#### 2.2.3 Weight test

This study was performed after training and adaptation. Given the motor affectation and muscle weakness observed in the DCM mouse model, the test was performed using only one weight of 13 g (Deacon, 2013). In brief, mice were held by the middle zone of the tail to allow them to properly grasp the 13 g weight. Time was measured from the time the animal held the weight until it dropped it. Data are expressed in seconds as the total time of the mouse holding the weight.

#### 2.2.4 Wire hanging test

Mice were allowed to grasp a wire of 3 mm thick metal between two vertical stands with the two forepaws. The vertical stands were 30 cm high (above the floor). The latency to fall of each animal was recorded and expressed in seconds (van Putten et al., 2012).

### 2.3 Spinal motoneurons and excitatory synaptic input counts

Spinal cord tissue was processed as previously reported (Tejero et al., 2020). The number of somatic proprioceptive inputs per somata and the number of motoneurons were stained and analyzed according to our previous study (Tejero et al., 2020). In brief, C5-C6 SCs tissue was fixed in 4% of paraformaldehyde (PFA) during 16 h, cryoprotected in 30% of sucrose/PBS 0.01 M, OCT-embedded (Sakura Fine Technical Company, Torrance, CA, United States), and cut in 30 μm thick cryosections. For motoneurons count and synaptic input quantification, SC slices were permeabilized with 0.5% Triton X-100/PBS 0.01 M for 20 min, washed in PBS 0.01 M, and blocked in 4% of BSA/Triton x-100/PBS 0.01 M. Slices were immunoassayed with the following primary antibodies: polyclonal goat anti-ChAT 1:150 (AB144P, Millipore), mouse anti-NeuN 1:1000 (MAB377, Millipore), and VGLUT1 1:100 (SYSY ref. 305,303) overnight (O.N) at 4°C. The following secondary antibodies were used: donkey anti-goat Cy3, donkey anti-mouse Cy5, and donkey anti-rabbit Alexa-488 (all of them were donkey H + L, Jackson Immunoresearch Laboratories, West Grove, PA, United States), all at 1:400 dilution. Analysis was done in serial slices, each 10th section covering a total SC segment of at least 3 mm. Imaging acquisition was performed on an LSM700 and LSM780 (Zeiss) confocal microscope with a 40x oil-immersion objective (N.A.:1.3), and the analysis was performed with ImageJ software. The number of motoneurons (NeuN^+^/ChAT^+^) was counted in 10 slices, while the VGLUT1^+^ and ChAT^+^ synapses were counted at the epicenter of the damage between the 4^th^ and 6^th^ sections. The compression ratio of the SC was examined by measuring the ratio between the anteroposterior SC diameter versus the transversal SC diameter and normalized (Ahn et al., 2010). The cross-sectional area (CSA) of the SC segment at the epicenter of the damage (4th or 5th SC section) was measured concerning the total area (white and gray matter).

### 2.4 Muscle fiber immunohistochemistry

Mice were euthanized, and the muscle biceps brachii (BB) and supraspinatus were processed by immunohistochemistry (IHC) as previously described (Woehlbier et al., 2016; Ojeda et al., 2020; Tejero et al., 2020). In brief, tissue was fixed with 4% PFA for 90 min, OCT-embedded and sectioned every 20 μm, and collected on coated slides (PC2-302-08, PorLab). Cryosections were stained with an NADH-reduced solution (Tris-buffer, pH 7.4, NADH reduced, nitro-blue tetrazolium; Sigma Aldrich, St. Louis, MO, United States) for 45 min at RT, and fibers were classified into slow (dark), intermediate (gray), or fast (white). The identity of all muscle fibers contained within the BB muscles and the cross-sectional area (CSA) of >1,200 fibers per type in each BB muscle was determined using the ImageJ software. To calculate CSA and regeneration, the muscle membrane and nuclei were stained with the wheat germ agglutinin lectin (WGA, Molecular Probes, Waltham, MA, United States (1 μg/mL) conjugated to Alexa 488) plus DAPI (Molecular Probes, MA, United States, 1 μg/mL) for 20 min (Woehlbier et al., 2016; Tejero et al., 2020). Given that we have observed that the DCM mouse model lost skeletal muscle fibers after 2 months of injury compared with the control group, a number of central nuclei were normalized by the total transversal area of each muscle.

### 2.5 Neuromuscular junction staining, imaging, and analysis

Skeletal muscle supraspinatus innervated by C5-6 spinal cord segments were stained, as previously reported (Woehlbier et al., 2016; Ojeda et al., 2020; Tejero et al., 2020). First, muscle tissue was fixed in PFA 0.5% for 90 min, cryoprotected in 30% of sucrose PBS 0.01 M, and OCT-embedded and cut in slides of 200 μm. Slides were washed in 0.15 M of glycine for 30 min, permeabilized with 1% non-ionic detergent Triton X-100 for 1 h, and incubated in 4% BSA in PBS 0.01 M for 3 h at RT (blocking solution). Primary antibodies, mouse anti-SV2 (DSHB, 1:100)/mouse anti-SMI32 (1,750, Covance ref. SMI-32P), were incubated O.N at 4°C for 16 h in blocking solution. Afterward, samples were washed in PBS 0.01 M with 0.05% Triton X-100 for 1 h and incubated with the secondary antibody donkey anti-mouse Cy3 1:300 (Jackson Immunoresearch Laboratories) and 10 ng/mL rhodamine-BTX (T0195, Thermofisher) for 1 h. During the last step, samples were washed and mounted with a fluorescence medium (Sigma). Images were acquired using a Zeiss LSM 700 laser scanning confocal microscope (CMA BioBio, Universidad de Concepción, Concepción, Chile). Confocal z-plane optical sections (1 μm) were captured using 40× (Plan-Apochromat 40×/1.3 Oil DIC M27) and 63× (Plan-Apochromat 63×/1.40 Oil DIC M27) objectives. The area of the presynaptic motor terminal within the NMJ region and the total AChR positive area of >40 NMJs per mouse were calculated (Jones et al., 2016). Data are presented as the area (μm^2^), perimeter (μm), and length (μm) of the major and minor axis of the presynaptic motor axon terminal and the postsynaptic AChR clustering.

### 2.6 Colon staining and analysis

The proximal and distal areas of the colon were dissected out and fixed in 10% PFA. OCT-embedded sections of 20 μm were stained overnight (O.N) at 4°C with mouse anti-E-Cadherin (Santa Cruz ref. sc-21791, 1:200) for immunohistochemistry characterization. After washing with PBS 0.01 M, the secondary antibody donkey anti-mouse Cy5 (Jackson Immunoresearch Laboratories, 1:400) was incubated for 2 h at room temperature. For lectin immunohistochemistry, WGA-Alexa488 1 μg/mL (Molecular Probes, Waltham, MA, United States) and DAPI 1 μg/mL (Molecular Probes, MA, United States) were incubated for 20 min before rinsed and mounted with fluorescence medium (Sigma). A tilt scan was done in an LSM780 (Zeiss) microscope, and the results of the total area (μm^2^) of mucin granules were quantified using the Otsu threshold (Otsu, 1979) of ImageJ since it is an option with excellent performance at different size of particles and in multi-intensity. Then, the results were normalized by the total mucosa layer (manually selected to compensate for the variation in the cross-sectional area of the colon).

### 2.7 NSC34 culture, differentiation, and treatments

The neuroblastoma x spinal cord NSC34 cells (Cashman et al., 1992) were grown in Dulbecco’s modified Eagle’s medium (DMEM, Gibco) supplemented with 10% fetal bovine serum and 1% penicillin/streptomycin solution at 37°C in a 5% CO_2_ atmosphere. Plastic or glass surfaces were coated for 1 h with vitronectin, and 40.000 cells were seeded to differentiate after 24 h in the Neurobasal medium (Gibco). The culture medium was changed every other day for 72 h. Treatment with acid acetate, butyrate, and propionate (200 μM) was performed for 72 h daily (Ribeiro et al., 2020). For immunohistochemistry, NSC34 cells were fixed in 4% PFA for 20 min, permeabilized with 0.5% Triton X-100/PBS 0.01 M for 15 min, and blocked in BSA 4%/PBS 0.01 M for 1 h at RT. Primary antibodies, mouse anti-b-actin (Santa Cruz, 1:500) and rabbit anti-β-III tubulin (Sigma ref. T3952, 1:1000), were incubated for 16 h in a blocking solution. Corresponding Cy2 conjugated secondary antibodies (Jackson Immunoresearch Laboratories, 1:300) and 30 μM DAPI (Molecular Probes, MA, United States) were incubated for 2 h at RT. Afterward, samples were washed and embedded with a Fluoromount fluorescence medium. Images were captured in a Nikon microscope with a ×40 Air Plan Achromat Objective (N.A.: 0.65), and the mean of neurite length (μm) quantification was done in motoneurons >500 per condition from three independent experiments using ImageJ.

### 2.8 Microbial DNA purification, 16S amplicon preparation, sequencing, and processing

Fecal samples were collected and sent to Microbiome Insights (Canada) for 16S sequencing. In brief, specimens were placed into a MoBio PowerMag Soil DNA Isolation Bead Plate. DNA was extracted on a KingFisher robot following MoBio’s instructions. Bacterial 16S rRNA genes were PCR-amplified with dual-barcoded primers targeting the V4 region (515F 5’-GTGCCAGCMGCCGCGGTAA-3′ and 806R 5’-GGACTACHVGGGTWTCTAAT-3′), as per the protocol of Kozich et al. (2013). Amplicons were sequenced with an Illumina MiSeq using the 300-bp paired-end kit (v.3). Sequences were denoised, taxonomically classified using Silva (v. 138) as the reference database, and clustered into 97%-similarity operational taxonomic units (OTUs) with the mothur software package (v. 1.44.1; Schloss et al., 2009), following the recommended procedure. The number of samples analyzed was four per condition, and data are shown as the Firmicutes: Bacteroidetes ratio.

## 3 Results

### 3.1 Diminished motor performance after DCM

**Figure 1:**
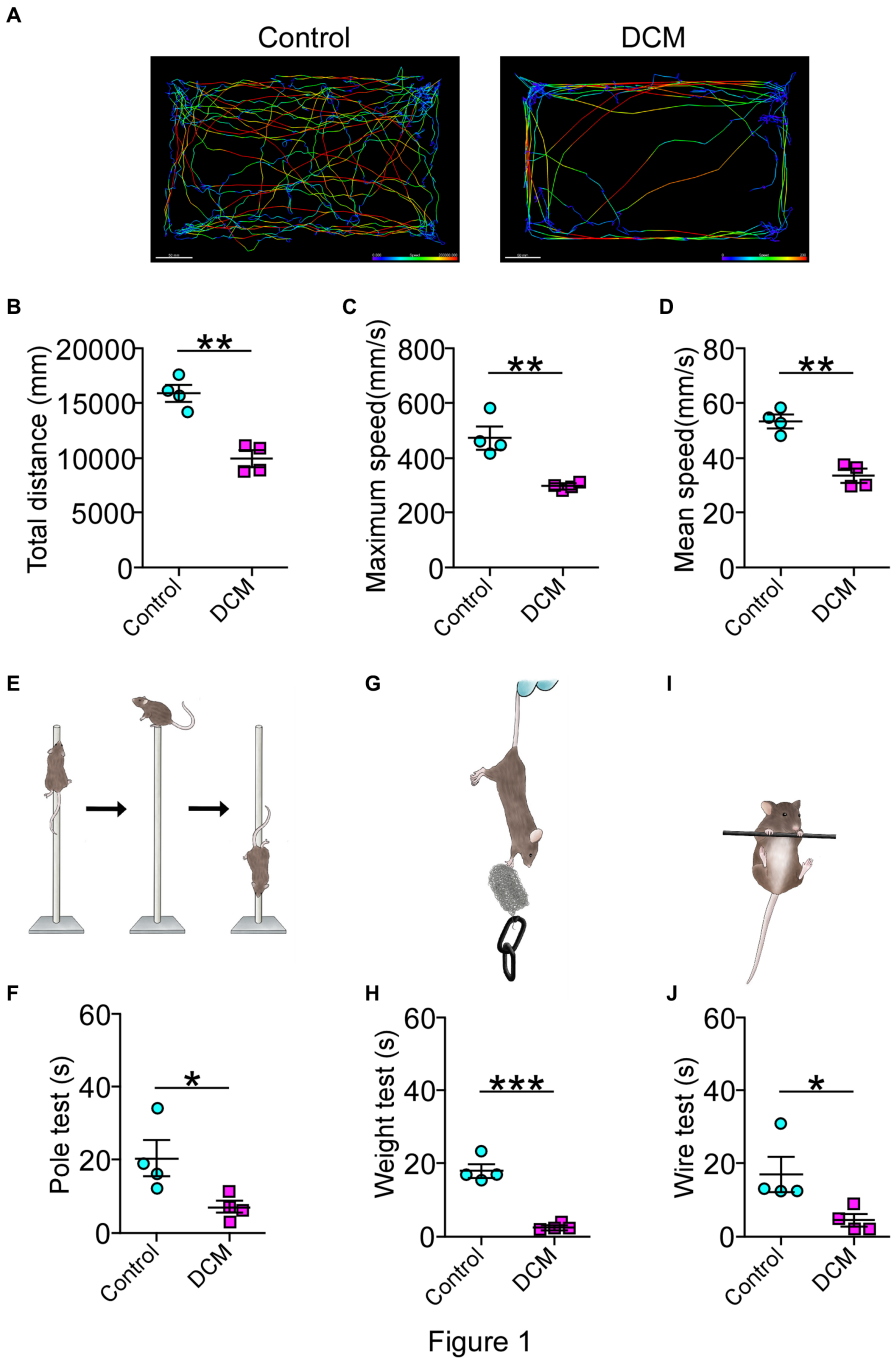
Diminished motor performance after DCM. Male DCM mice and their control mice were challenged to different motor tests abilities. **(A)** An illustrative example of the heatmap travel path during 5 minutes on the open field test to control and DCM mouse (scale bar: 50 mm). DCM mice were less active as measured in **(B)** total distance (control: 15844 ± 709 mm; DCM: 9904 ± 712 mm; *p* < 0.001 *t*-test; **p < 0.001), **(C)** maximum (control: 470.8 ± 39.4 mm/s; DCM: 294.3 ± 9.3 mm/s; **p < 0.0048 *t*-test) and **(D)** average (control: 52.8 ± 2.3 mm/s; DCM: 33.1 ± 2.4 mm/s; **p < 0.0012 *t*-test) speed. In turn, **(E,F)** pole test (control: 22.5 ± 3.7 s, DCM: 5.7 ± 0.4 s; *p < 0.0042 *t*-test, bar 8 mm), **(G,H)** weight test (control: 13.6 ± 2.9 s; DCM: 2.5 ± 0.5 s; ***p < 0.0071 *t*-test), and the **(I,J)** wire hang test (control: 17.0 ± 4.6 s; DCM: 4.5 ± 1.6 s; *p < 0.0452 t-test) confirm the motor deficit in the DCM group. Data are presented as mean ± s.e.m.; *n* = 4 per group.

For the DCM mouse model used in this study, the onset of the pathology was evidenced as early as the first month of polymer implantation through the emergence of a motor phenotype and the development of symptoms monitored over time until 12 weeks, as previously reported (Vidal et al., 2017; Ulndreaj et al., 2022). To further and more specifically study the DCM model, a battery of locomotor tests, including open field test, pole test, wire test, and weight test, were conducted 12 weeks after DCM induction. Remarkably, the open field test evidenced a dramatic change affecting the locomotor pattern of the DCM group compared with the control group (Figure 1). Specifically, DCM mice exhibited a 1.5-fold decrease in the average traveled total distance (control: 15844 ± 709 mm; DCM: 9904 ± 712 mm; ^**^p < 0.001 *t*-test; Figures 1A,B), the maximum velocity (control: 470.8 ± 39.5 mm/s; DCM: 294.3 ± 9.3 mm/s; ^**^p < 0.0048 *t*-test; Figure 1C), and the average displacement velocity (control: 52.9 ± 2.4 mm/s; DCM: 33.2 ± 2.5 mm/s; ^**^p < 0.0012 *t*-test; Figure 1D). Furthermore, the DCM group spent more time immobile (control: 54.2 ± 2.5%; DCM: 72 ± 3.0%; ^**^p < 0.0039 *t*-test; Supplementary Figure S1A), primarily at the edges of the box (control: 48.8 ± 2.3%; DCM: 77.75 ± 6.8%; ^**^p < 0.0063 *t*-test; Supplementary Figure S1B), particularly in the corners (control: 28.5 ± 2.5%; DCM: 48.5 ± 6.4%; ^*^p < 0.028 *t*-test; Supplementary Figure S1C). The DCM group also showed reduced time spent in the center of the box (control: 66 ± 2.4%; DCM: 22.2 ± 6.6%; ^***^p < 0.0008 *t*-test; Supplementary Figure S1D), highlighting the altered locomotor behavior in the DCM group compared with the control group. Consistently, the pole test showed that the DCM group descended along the pole much faster than the control group, with a significant reduction in descending the time (DCM: 5.8 ± 0.4 s, control: 22.5 ± 3.7 s; ^*^p < 0.049 *t*-test; Figures 1E,F). This effect was also observed with two other diameters of the pole included to discard cognitive alterations with a significant difference for 6 mm (control: 20.25 ± 4.8 s; DCM: 6.8 ± 1.6 s; ^**^p < 0.021 *t*-test; Supplementary Figure S1E) and 10 mm (control: 35.2 ± 4.8 s; DCM: 7.0 ± 0.6 s; ^**^p < 0.0012 *t*-test; Supplementary Figure S1F). We extended the analysis of motor performance challenging mice with a more demanding task by using the principal motor circuit affected by testing their forelimb muscle strength in the weight and wire tests. The evaluation of motor capability to hold 13 g (control: 13.6 ± 2.9 s; DCM: 2.5 ± 0.5 s; ^***^p < 0.0071 *t*-test; Figures 1G,H) or its own weight (control: 17.0 ± 4.6 s; DCM: 4.5 ± 1.6 s; ^*^p < 0.0452 *t*-test; Figures 1I,J) was strongly impaired in the DCM group compared with the control. Thus, these results evidence a strong motor decline of upper limbs after DCM.

### 3.2 The DCM mouse model exhibits remarkable histopathological alterations at the compression level

**Figure 2:**
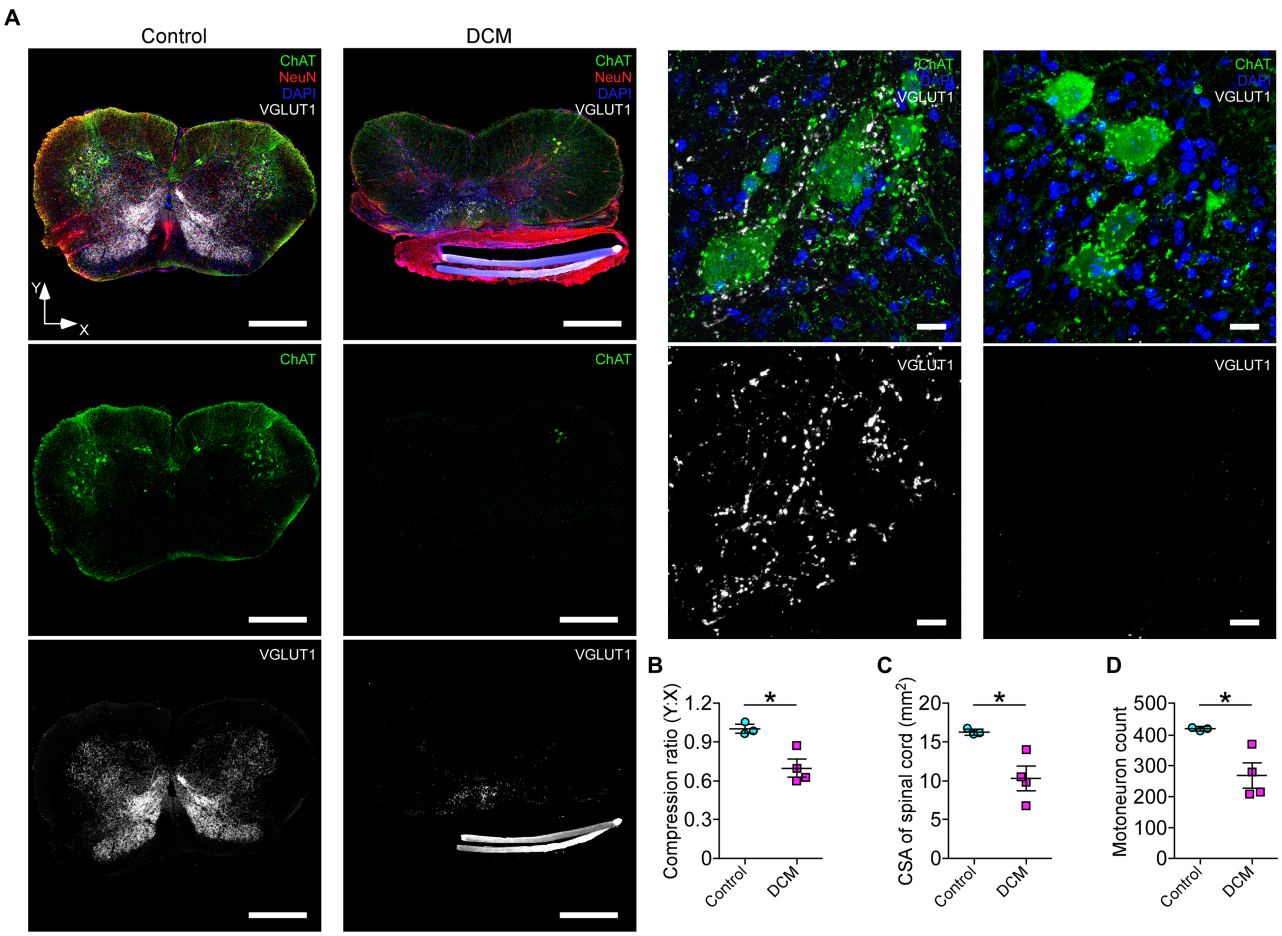
The DCM mouse model presents remarkable histopathological alterations at the compression level. **(A)** Representative slices of C5-6 spinal cord transversal sections from control and DCM group were stained against choline acetyltransferase (ChAT; green), vesicular glutamate transporter 1 (VGLUT1; white), NeuN neuronal marker (red), and nuclei (DAPI; blue) to show the cellular organization and distribution of glutamatergic excitatory synaptic inputs (scale bar: 500 μm). (A, low panel). Zoomed images of control and DCM from the ventral horn show that motoneurons present cholinergic (green dots) and glutamatergic (white dots) synaptic inputs (scale bar: 25 μm). Graphs express the results of the **(B)** ratio between the antero-posterior (Y) and transversal (X) spinal cord length (control: 1.0 ± 0.03; DCM: 0.69 ± 0.06; ^*^p < 0.0152 *t*-test), the **(C)** cross-sectional area of the spinal cord by the slice (control:16.2 ± 0.3 mm^2^; DCM: 10.3 ± 1.5 mm^2^; ^*^p < 0.024 *t*-test), and the **(D)** number of motoneurons counted in 10 serial SC slices (control: 419 ± 6; DCM: 267 ± 39; ^*^p < 0.023 *t*-test). Data are presented as mean ± s.e.m. and statistical analysis was performed using unpaired *t*-test; *n* control = 3, *n* DCM = 4.

**Figure 3:**
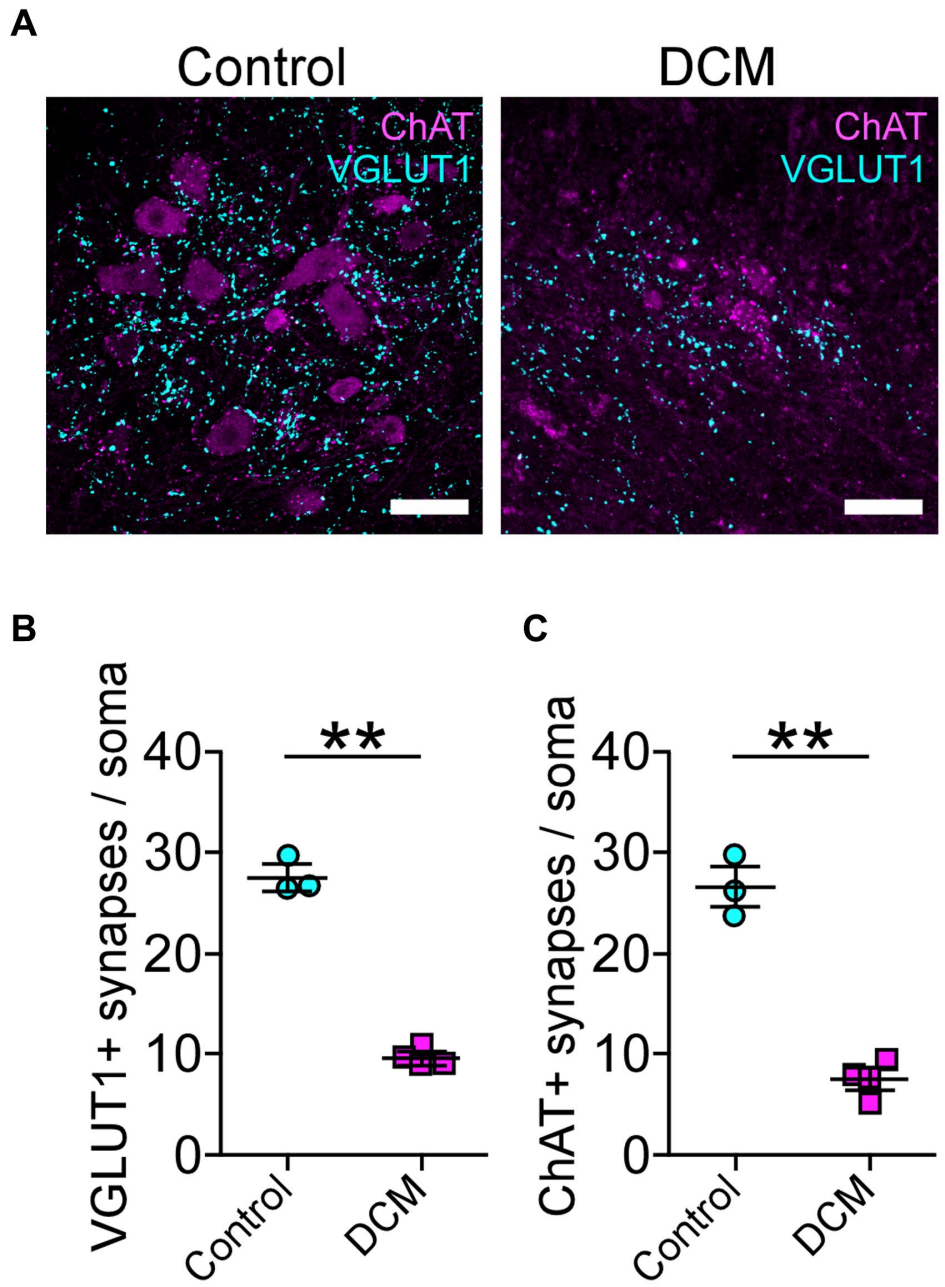
Decreased cholinergic and glutamatergic excitatory synaptic inputs in the cervical spinal cord neurons after DCM. **(A)** Control and DCM mice were stained using antibodies against ChAT (magenta) and VGLUT1 (cyan), as is shown in the representative images (scale bar: 100 μm). Quantification of **(B)** VGLUT1-positive (control: 24 ± 2; DCM: 10 ± 1; ^**^p < 0.0031 *t*-test) and **(C)** ChAT (control: 26 ± 3; DCM: 8 ± 1; ^**^p < 0.0036 *t*-test) spinal neurons evidence a decrease of both synaptic inputs. Data are presented as mean ± s.e.m.; *n* control = 3, *n* DCM =4.

Polymer implantation in C5-6 segments causes progressive stenosis at the central canal, triggering changes in the vasculature and the immune response, among other phenotypes (Karadimas et al., 2013; Vidal et al., 2017; Ulndreaj et al., 2022). We examined in detail the SC histology focusing on motoneurons as a read-out of motor disorders (Wilson et al., 2023; Figure 2A). After 12 weeks of DCM induction, the immunohistochemical analysis showed important structural changes with a reduction in the anteroposterior versus transversal diameter ratio (control: 1.0 ± 0.03; DCM: 0.69 ± 0.06; ^*^p < 0.0152 *t*-test; Figure 2B). In turn, the cross-sectional area of the spinal cord slices (Figure 2C) from the C5-6 laminae was decreased by approximately 5.94 mm^2^ in the DCM group compared with the control group (control:16.2 ± 0.3 mm^2^; DCM: 10.3 ± 1.5 mm^2^; ^*^p < 0.024 *t*-test; Figure 2C). Remarkably, the immunofluorescence of double positive cells stained against choline acetyltransferase (ChAT) and the neuronal nuclear (NeuN) proteins (Figure 2A, green channel) indicate a 36.20% reduction in the total number of motoneurons counted in the C5-6 area (control: 419 ± 6; DCM: 267 ± 39; ^*^p < 0.023 *t*-test; Figure 2D). Moreover, to analyze the synaptic effects on the ventral motoneurons caused by the compressive stenosis on the dorsal horns, a number of excitatory somatodendritic inputs were also analyzed in both groups (Figure 3A). The results indicate a significative decrease in the number of VGLUT1-positive inputs per motoneuron soma when the spinal cord was compressed (control: 24 ± 2; DCM: 10 ± 1; ^**^p < 0.0031 *t*-test; Figure 3B). Thus, DCM led to a 50% reduction of VGLUT1-positive inputs per motoneuron soma compared with the control group. ChAT-positive inputs on the motoneuron somata (ChAT and NeuN double-positive motoneurons) were also counted, and we found a 3.25-fold decrease in the number of cholinergic synapses per motoneuron soma (control: 26 ± 3; DCM: 8 ± 1; ^**^p < 0.0036 *t*-test; Figure 3C). Altogether, our findings showed cellular and synaptic changes occurring within the spinal cord after DCM.

### 3.3 DCM causes muscle and neuromuscular impairment

**Figure 4:**
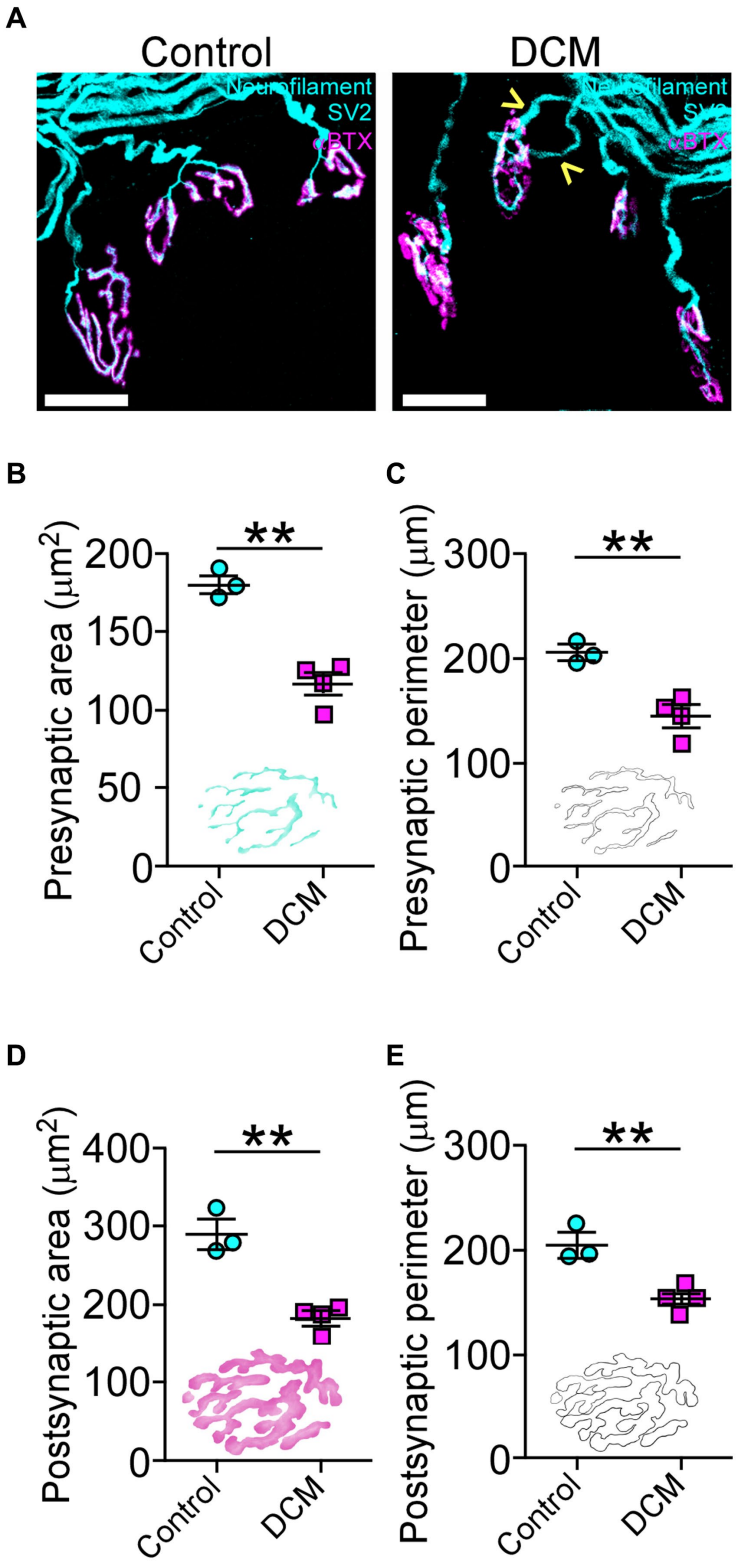
DCM causes neuromuscular impairment. **(A)** Whole mounted supraspinatus muscles were dissected and stained against neurofilament/SV2 (cyan) and αBTX (magenta; scale bar: 25 μm). The quantification of the morphometric analysis for the **(B)** area (control: 179.8 ± 5.0 μm^2^; DCM: 116.3 ± 7.0 μm^2^; ^**^p < 0.0004 *t*-test) and **(C)** perimeter (control: 204.5 ± 6.9 μm; DCM: 143.5 ± 10.3 μm; ^**^p < 0.0063 *t*-test) of the presynaptic axon terminal and the **(D)** area (control: 288.9 ± 18.9 μm^2^; DCM: 181.0 ± 9.1 μm^2^; ^**^p < 0.0025 *t*-test) and **(E)** perimeter (control: 204.6 ± 11.8 μm; DCM: 152.9 ± 4.4 μm; ^**^p < 0.058 *t*-test) for the postsynaptic endplate indicate that NMJs of DCM mice are smaller in comparison with the control group. Data are presented as mean ± s.e.m.; *n* control = 3, *n* DCM =4.

To determine whether DCM changes the structure of neuromuscular communication, we examined the supraspinous skeletal muscle, as it is mainly innervated by motoneurons from the compressed SC region (Bácskai et al., 2013). The presynaptic proteins were analyzed using markers for neurofilament and synaptic vesicles (SV2), while the postsynaptic AChRs were visualized by labeling with α-bungarotoxin (αBTX; Figure 4A). After 12 weeks of DCM induction, NMJs appear fully innervated by motor axon terminals contacting postsynaptic muscle specializations as in control mice, despite the substantial reduction in the number of motoneurons (Figure 3). However, a certain level of poly-innervation was observed in some synaptic sites (arrowheads in Figure 4A, DCM). Structurally, the quantitative analysis of NMJs revealed that DCM resulted in smaller endplates with a significative decrease in the measurement of presynaptic area (control: 179.8 ± 5.0 μm^2^; DCM: 116.3 ± 7.0 μm^2^; ^**^p < 0.0004 *t*-test; Figure 4B) and perimeter (control: 204.5 ± 6.9 μm; DCM: 143.5 ± 10.3 μm; ^**^p < 0.0063 *t*-test; Figure 4C), as compared with the control group. There was also a decrease in the length of major (control: 34.9 ± 0.8 μm; DCM: 24.5 ± 3.1 μm; ^*^p < 0.048 *t*-test; Supplementary Figure S2B) and minor (control: 19.4 ± 0.8 μm; DCM: 13.1 ± 1.9 μm; ^*^p < 0.045 *t*-test; Supplementary Figure S2C) axes of the nerve terminal branches, although these structures maintained their aspect ratio (control: 2.1 ± 0.2; DCM: 2.3 ± 0.1; *p* < 0.52 *t*-test; Supplementary Figure S2D) and roundness (control: 0.5 ± 0.03; DCM: 0.5 ± 0.02; *p* < 0.24 *t*-test; Supplementary Figure S2E). The analysis of confocal micrographs of immunolabeled postsynaptic structures revealed changes in size in the nerve terminal, implicating a high degree of congruence in the architecture of the NMJ. Specifically, the area (control: 288.9 ± 18.9 μm^2^; DCM: 181.0 ± 9.1 μm^2^; ^**^p < 0.0025 *t*-test; Figure 4D) and perimeter (control: 204.6 ± 11.8 μm; DCM: 152.9 ± 4.4 μm; ^**^p < 0.058 *t*-test; Figure 4E) of the postsynaptic AChR clusters was reduced, in line with a decreased length of the major (control: 34.9 ± 0.8 μm; DCM: 27.3 ± 2.3 μm; ^*^p < 0.0442 *t*-test; Supplementary Figure S2F) and minor (control: 20.0 ± 1.0 μm; DCM: 14.4 ± 1.2 μm; ^*^p < 0.0201 *t*-test; Supplementary Figure S2G) axes. Subsequently, the values of aspect ratio (control: 2.0 ± 0.1; DCM: 2.2 ± 0.1; *p* < 0.0369 *t*-test; Supplementary Figure S2H) and roundness factor (control: 0.57 ± 0.02; DCM: 0.53 ± 0.03; *p* < 0.3911 *t*-test; Supplementary Figure S4I) were similar between control and DCM groups. Overall, the supraspinous muscle, affected by DCM, exhibits a markedly impaired architecture of NMJs.

**Figure 5:**
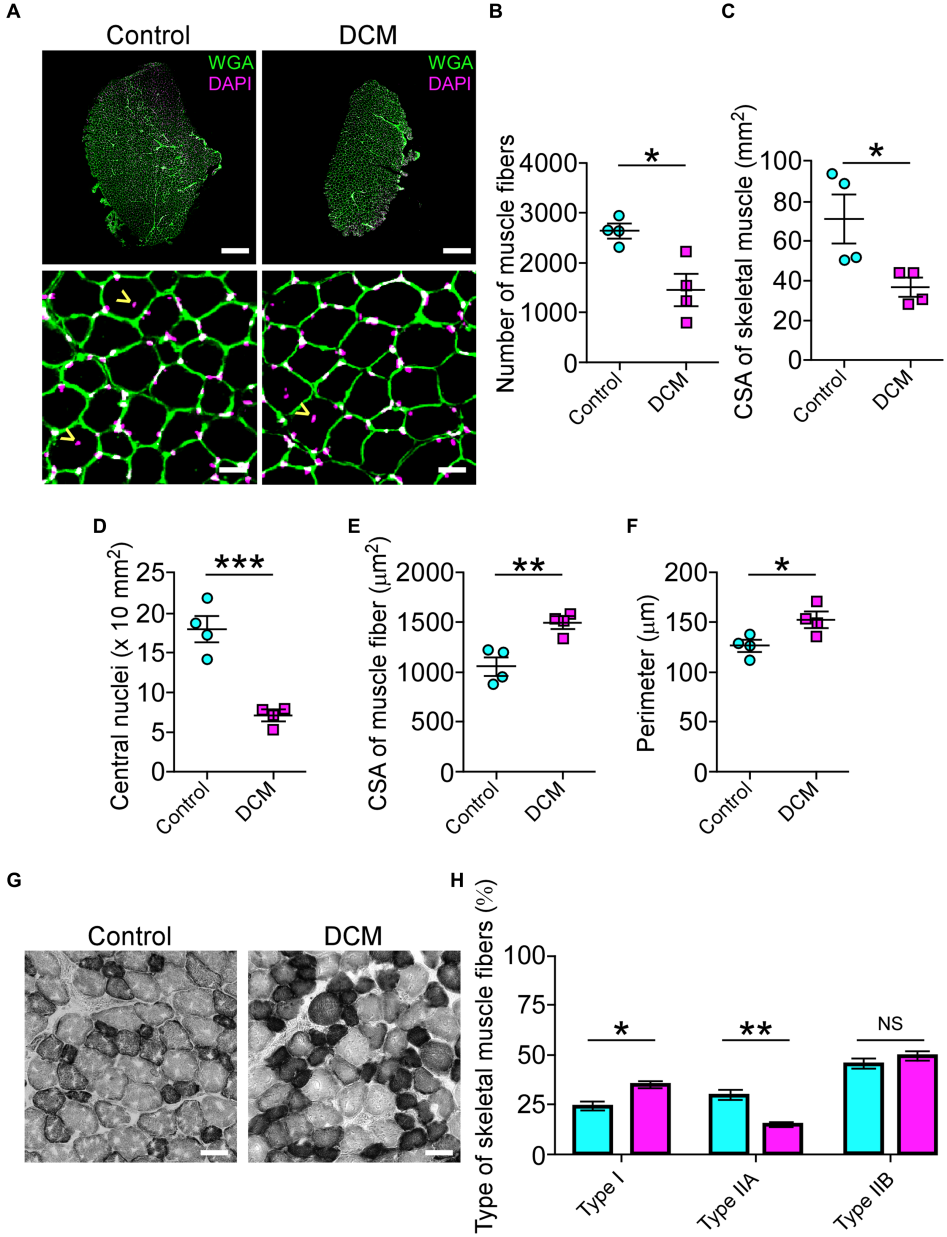
DCM impairs skeletal muscle tissue. **(A)** Cross sections of 20 μm from Control and DCM mice were stained against lectins with Alexa488-WGA (green) and nuclei with DAPI (magenta; scale bar: 500 μm and 25 μm, respectively). **(B)** The number of skeletal muscles fibers (control: 2601 ± 140; DCM: 1427 ± 309; ^*^p < 0.0135 *t*-test) as well as the **(C)** CSA of skeletal muscle (control: 70.3 ± 12 mm^2^; DCM: 36.1 ± 4.6 mm^2^; ^*^p < 0.0386 *t*-test) and the **(D)** number of central nuclei (control: 18 ± 2; DCM: 7 ± 1; ^***^p < 0.0008 *t*-test) as a parameter of muscle fiber damage/regeneration, the **(E)** CSA of skeletal muscle fibers (control: 1062 ± 95; DCM: 1497 ± 61; ^**^p < 0.0086 *t*-test) and its **(F)** perimeter (control: 126.1 ± 6.2 μm; DCM: 152.0 ± 7.9 μm; ^*^p < 0.042 *t*-test) were quantified. **(G)** Transversal cryosections stained with NADH-TR histochemical activity detection were used to analyze fast (light and big), intermediate (dark and big), and slow (dark and small) fibers. **(H)** The proportion of these fiber types was quantified and expressed as a percentage of total fibers of the muscles (control - DCM, type I: 24.7 ± 1.5–35.0 ± 3.9; type IIA: 29.8 ± 4.8–15.2 ± 2.3; type IIB: 45.4 ± 5.2–49.6 ± 5.1; ^*^p < 0.02 and ^**^p < 0.0011 *ANOVA* with *Tukey’s* correction). The results represent the mean ± s.e.m. of *n*: 4 mice per group.

The muscle performance revealed a markedly decreased motor capacity (Figure 1; Supplementary Figure S1). The changes in the morpho-architecture of NMJs innervated by motoneurons located in the compressed region of the SC suggested that skeletal muscle cells might be responsible for the neuromotor damage. Based on our findings showing decreased motor performance (Figure 1; Supplementary Figure S1), we next explored the consequences of DCM on histopathological features of the BB muscle, also innervated by motoneurons from the zone of chronic compression. First, we aimed to determine the number of skeletal muscle fibers on muscle transversal cryosections (Figure 5). A gross reduction of the BB muscle in the DCM group compared with the control group was observed (Figure 5A). Of note, a similar effect was observed in other muscles (Supplementary Figure S3A). Quantitative histopathological analyses revealed a 1.8-fold reduction of the number of total skeletal muscle fibers (control: 2601 ± 140; DCM: 1427 ± 309; ^*^p < 0.0135 *t*-test; Figure 5B) that was concomitant with a 1.9-fold decrease in the cross-sectional area of the BB muscle (control: 70.3 ± 12 mm^2^; DCM: 36.1 ± 4.6 mm^2^; ^*^p < 0.0386 *t*-test; Figure 5C). Furthermore, we found a 2.6-fold decrease in the number of central nuclei in the DCM group, compared with the control group, suggesting an impaired capability of muscle regeneration (control: 18 ± 2; DCM: 7 ± 1; ^***^p < 0.0008 *t*-test; Figure 5D). Interestingly, the remaining skeletal muscle fibers showed an increase in the parameters of cross-section area (control: 1062 ± 95 μm^2^; DCM: 1497 ± 61 μm^2^; ^**^p < 0.0086 *t*-test; Figure 5E) and perimeter (control: 126.1 ± 6.2 μm; DCM: 152.0 ± 7.9 μm; ^*^p < 0.042 *t*-test; Figure 5F), likely related to an adaptative mechanism for the loss of muscle fibers. Detailed analyses of size frequency distribution per muscle indicate that the number of myofibers having a CSA below 1,200 μm^2^ was dramatically reduced in the DCM group with the subsequent increase in the transversal area of skeletal muscle fiber having a CSA over 1800 μm^2^ (Supplementary Figure S3B), as compared with the control group (control - DCM, 0–599: 656 ± 105–116 ± 68; 600–1,199: 1005 ± 120–506 ± 209; 1,200–1799: 590 ± 70–506 ± 178; 1800–2,399: 240 ± 279–766 ± 160; ^*^p < 0.016 *two-way ANOVA*). To assess potential changes in the muscle fiber types with DCM, we performed histochemical labeling to quantify NADH-thioreductase activity (Figure 5G). In terms of metabolism and mitochondria number, we considered the following classification of myofibers: fast-twitch type I (non-oxidative, light gray, and bigger CSA), fast-twitch type IIA (oxidative, middle gray, and bigger CSA), and slow-twitch type IIB (oxidative, dark gray, and smaller CSA). Our analysis showed that DCM resulted in myofiber plasticity expressed as changes in the percentage of muscle fiber types by increasing the muscle fibers type I at the expense of a reduction in the type IIA when compared with the control group (control - DCM, type I: 24.7 ± 1.5–35.0 ± 3.9; type IIA: 29.8 ± 4.8–15.2 ± 2.3; type IIB: 45.4 ± 5.2–49.6 ± 5.1; ^*^p < 0.02 and ^*^p < 0.0011 *ANOVA* with *Tukey’s* correction; Figure 5H). Thus, these findings show that DCM results in muscle impairment by inducing muscular atrophy and metabolic adaptation. Indeed, our findings underscore a significant disruption in the peripheral communication between motoneurons and muscles, driving the comprehension of the pivotal role these direct mechanisms play in the pathobiology of muscle weakness. This insight holds great promise for advancing our efforts to ameliorate the manifestations of DCM and alleviate its associated symptoms.

### 3.4 Cervical spinal cord compression leads to disruption of the intestinal mucosal barrier

**Figure 6:**
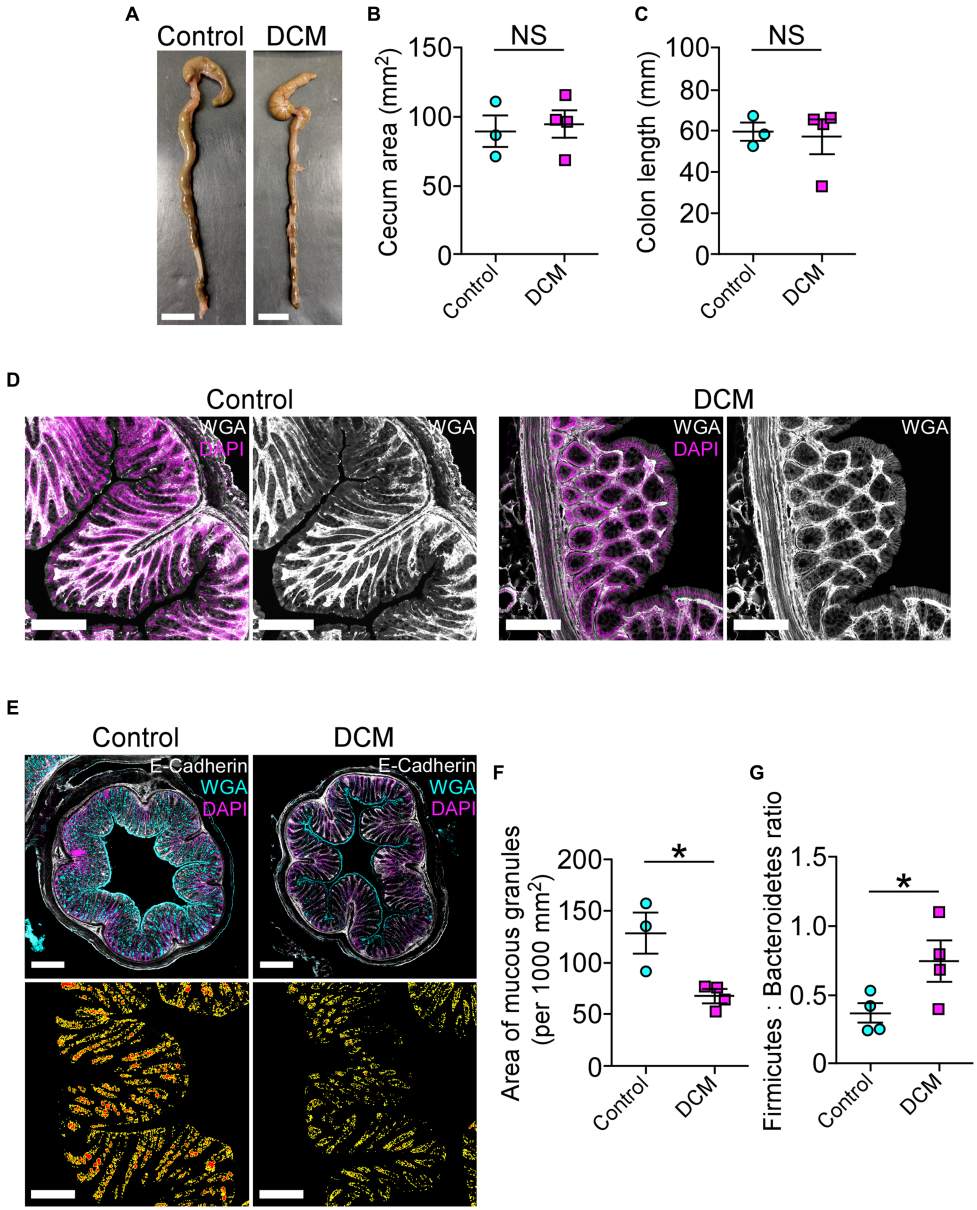
The cervical spinal cord compression leads to disruption of the intestinal mucosal barrier. **(A)** Whole colon tissue (scale bar 10 mm) was examined from control and DCM mice, and the **(B)** cecum area (control: 89.2 ± 11.7 mm^2^; DCM: 94.5 ± 9.6 mm^2^; *p* < 0.74 *t*-test) and the **(C)** colon length (control: 59.6 ± 4.5 mm; DCM: 57.2 ± 8.2 mm; *p* < 0.82 *t*-test) size were analyzed in both groups. **(D)** Representative images of three mice per group show the integrity of the intestinal epithelial barrier in cross-sectional views at the proximal segment to the anus by staining against E-cadherin (white) and DAPI (magenta; scale bar: 200 μm). **(E)** Transversal sections of the colon (scale bar: 500 μm) from control and DCM stained with Alexa488-WGA (cyan) to detect mucous granules, E-cadherin antibody (white), and DAPI (magenta) reveal a reduced **(F)** area of mucous granules (cyan) at the mucosal layer (low panel in E, area and perimeter of granules in red and yellow, respectively; control: 128.2 ± 19.6 mm^2^; DCM: 67.4 ± 6.3 mm^2^; ^*^p < 0.02 *t*-test). **(G)** The phylum profile ratio related to the relative abundance of Firmicutes and Bacteroidetes was quantified (control: 0.37 ± 0.07%; DCM: 0.74 ± 0.1%; ^*^p < 0.043 *t*-test). Data are presented as mean ± s.e.m.; *n* control = 3, *n* DCM = 4 for **B**, **C**, and **F**; *n* control = 4, *n* DCM = 4 for **G**.

Accumulating evidence has shown that spinal cord pathologies are frequently accompanied by neurogenic intestinal dysfunction as a consequence of changes in motor control (Gungor et al., 2016; Zhang et al., 2018; Lin et al., 2020; Farini et al., 2023). On the other hand, the motor phenotype of patients and animal models with traumatic and non-traumatic SCI could be exacerbated by colon neuroinflammation, motility malfunction, and dysbiosis, among others (Gungor et al., 2016; Kundu et al., 2017; Zhang et al., 2018; Cryan et al., 2019; Lin et al., 2020). In the DCM mouse model, there were no significant differences in the cecum area (control: 89.2 ± 11.7 mm^2^; DCM: 94.5 ± 9.6 mm^2^; *p* < 0.74 *t*-test; Figures 6A,B) and in the colon length size (control: 59.6 ± 4.5 mm; DCM: 57.2 ± 8.2 mm; *p* < 0.82 *t*-test; Figure 6C) compared to the control group. Next, we evaluated the intestinal mucosal barrier integrity through immunostaining using antibodies directed against the tight junction protein E-cadherin, one of the most important structural gut components responsible for constitutive barrier function in epithelial cells (Schnoor, 2015). After 12 weeks of DCM induction, we did not observe differences in the E-cadherin signal localization at the border of the epithelial cells between the control and DCM groups (Figure 6D). However, a quantitative analysis of confocal fluorescence revealed smaller granules of mucin produced by goblet cells in the DCM group compared with the control group (Figure 6E). Specifically, the total area of mucous granules was 1.9-fold reduced in the DCM group compared with the control group (control: 128.2 ± 19.6 mm^2^; DCM: 67.4 ± 6.3 mm^2^; ^*^p < 0.02 *t*-test; Figure 6F). Under the same experimental conditions, we examined the phylum profile of Firmicutes and Bacteroidetes of gut microbiota based on its relative abundance in feces, as this parameter has been used as a marker of gut dysbiosis in different pathologies (Rowin et al., 2017; Mazzini et al., 2018; Blacher et al., 2019; Ngo et al., 2020; Stojanov et al., 2020; An et al., 2023). We observed that the ratio between Firmicutes and Bacteroidetes increased 2-fold in the DCM group compared to the control group (control: 0.37 ± 0.07%; DCM: 0.74 ± 0.14%; ^*^p < 0.043 *t*-test; Figure 6G). Therefore, our results suggest that DCM negatively alters the mucosa barrier and subsequently the gut microbiota balance.

### 3.5 Gut microbiota-derived metabolites influence the differentiation of the NSC-34 motoneuron cell line

**Figure 7:**
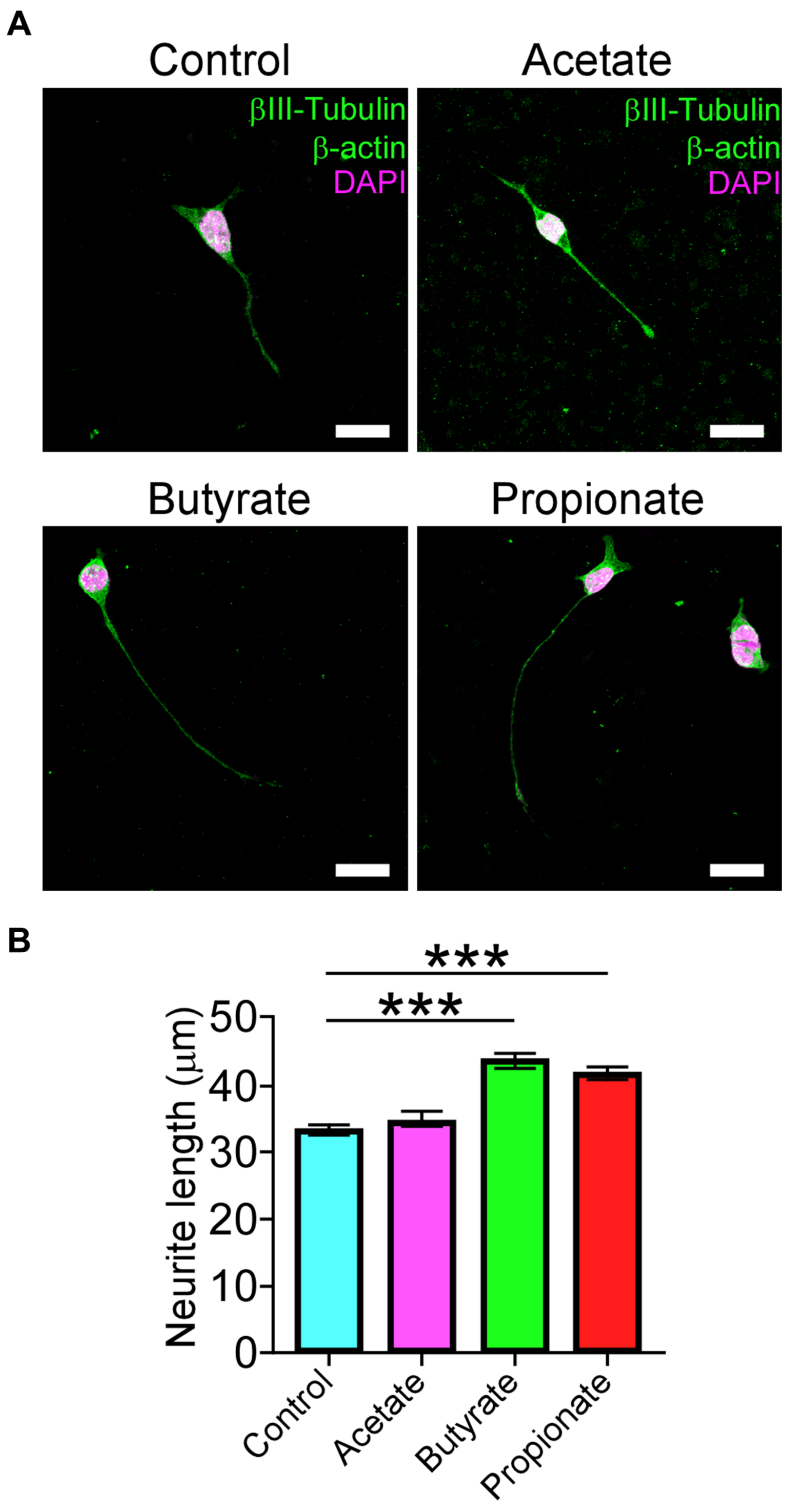
SCFAs promote the differentiation of motoneuron-like NSC34 cell lines. NSC34 cells were seeded on vitronectin and differentiated for 72 h. Fresh treatments with 200 μM of vehicle, acetate, butyrate, and propionate were performed every 12 h until the time of differentiation. After treatment, the differentiated cells were fixed in PFA 0.5% and stained against βIII-tubulin, β-actin, and DAPI, as shown in the **(A)** representative images. Quantification of **(B)** neurite length is shown (control: 33.2 ± 0.7 μm; acetate: 34.6 ± 1.0 μm; butyrate: 43.6 ± 1.2 μm; propionate: 41.7 ± 1.1 μm; ^***^p < 0.0001). Data are presented as mean ± s.e.m.

In agreement with the current progress of motoneuron disorders-induced gut dysbiosis, and given that our results showed an increased Firmicutes to Bacteroidetes ratio after DCM, we evaluate the potential effect of the most common short-chain fat acids (SCFAs), acetate, butyrate, and propionate, on the ability to differentiate motoneurons. Since altered Firmicutes:Bacteroidetes ratio has been associated with a reduction of SCFAs (Stojanov et al., 2020), we used the neuroblastoma x spinal cord NSC34 cells (Cashman et al., 1992) that present morphological and physiological properties of motoneuron-like cells *in vitro*. After supplementation with 200 μM of SCFAs every 12 h for 72 h, we fixed the cells and stained them against the βIII-tubulin and α-actin proteins for morphological quantification (Figure 7A). Surprisingly, butyrate and propionate treatments significantly increased the proportion of differentiated NSC34 cells projecting neurites compared with the control (control: 33.2 ± 0.7 μm; acetate: 34.6 ± 1.0 μm; butyrate: 43.6 ± 1.2 μm; propionate: 41.7 ± 1.1 μm; ^***^p < 0.0001 *ANOVA* with *Bonferroni* post correction; Figure 7B). Therefore, DCM could influence the colon mucosal barrier and the gut microbiota to affect the synthesis and/or secretion of possible gut-derived metabolites required for the normal maintenance of neuromotor communication.

## 4 Discussion

DCM, originating from various forms of central canal stenosis, emerges as a primary culprit behind motor impairments during adulthood (Badhiwala et al., 2020). The ongoing characterization of its pathogenesis at the cellular and molecular levels not only promises a deeper comprehension of this pathological presentation but also holds the potential to yield substantial insights into the broader landscape of motor neurodegenerative disorders. In this study, our results describe that after 12 weeks of cervical SC compression, the motor disabilities are severe, as evidenced by deteriorated locomotor performance and dramatic fore and hindlimb weakness. The observed motor deficits are concomitant with alterations in the cytoarchitecture of the SC (i.e., diminished population of motoneurons and reduced somatodendritic inputs). Furthermore, DCM results in a substantial loss of muscle fibers and a decline in their regenerative capacity. Consequently, these findings reveal that the neuromotor axis is severely compromised both at the structural and functional levels. Remarkably, our results also demonstrate that DCM exerts a negative impact on the mucosal intestinal barrier, manifested through a decrease in mucin granule size and alterations of the intestinal microbiota, as revealed by an unbalanced Firmicutes and Bacteroidetes ratio. SCFA treatment, butyrate, and propionate promoted differentiation of the motoneuron cell line NSC34, suggesting that DCM-induced dysbiosis may exert an influence on the preservation of motoneurons by SCFAs.

Recently, it has been suggested that a combination of different factors, and not simply spinal cord compression, are involved in the development of DCM (Davies et al., 2022). In this regard, changes in the narrowness of the spinal canal, whether due to static or dynamic factors, associated with changes in the composition of the intervertebral discs shock absorber or triggered by traumatic events, can lead to a diverse array of compressive myelopathies. These conditions manifest various sensory and motor symptoms, underscoring the complexity and variability of their clinical presentation (White and Panjabi, 1988; Baptiste and Fehlings, 2006). Degenerative changes can be derived from any component of the vertebra, either intervertebral disc, vertebral body, uncinated processes, facet joints, neural arch, or ossification of the posterior longitudinal or flavum ligaments (Kawaguchi et al., 2016), increasing the background onset and progression of DCM. A potentially useful model of DCM is a naturally occurring mutation in the tiptoe-walking (ttw) mouse that exhibits spontaneous calcified deposits at the C1-2 vertebral segment, causing paresis, spasticity, chronic compression of the cervical SC and a decline in the number of anterior horn neurons (Uchida et al., 1998; Yu et al., 2009). In contrast, the biopolymer polyurethane elastomer Aquaprene C works like an expandable material made of water-absorbing polyurethane elastomer that increases its volume more than 200% in 24 h inducing acute damage, different from the progression of the human pathology (Kim et al., 2004; Ijima et al., 2017). Here, we used a synthetic aromatic polyether polymer that offers clinical advantages to model DCM in mice, such as the ability to nucleate the precipitation of phosphate anions emulating the progressive osteoid formation in accessible vertebral segments of implantation, resembling histopathological and behavioral features of clinical presentation (Klironomos et al., 2011; Karadimas et al., 2013; Desimone et al., 2021; Laliberte et al., 2021; Ulndreaj et al., 2022). Using this model, we presented a systematic analysis of the pathobiology observed, including loss of motoneurons, motor impairment, and neuromuscular degeneration, as well as muscle loss and wasting (Otsu, 1979; Karadimas et al., 2013; Badhiwala et al., 2020). Interestingly, when evaluating the performance of DCM mice in attempting the same task with varying pole diameters, it becomes evident that their cognitive abilities remain unaffected despite the previously described sensory changes and neurological deficits (Karadimas et al., 2013; Laliberte et al., 2021).

The DCM mouse model has recently facilitated distinct *in vivo* approaches, shedding fresh insights into the neuronal mechanisms compromised. For instance, chronic SC compression initiates a cascade of events, including disruption of the microvasculature, reduction in the number of endothelial cells, increased apoptosis of neuronal and oligodendroglial cells, and the promotion of axonal degeneration in the proximal corticospinal segment subjected to the compression (Yu et al., 2011; Karadimas et al., 2013, 2015; Jiang et al., 2017). In this regard, the size of the spinal cord decreases in a wide range, extending rostrally and caudally, becoming severe at the injury epicenter, with the white and gray matter significatively reduced at the dorsal column. We detected the most pronounced neurodegeneration at the epicenter of the compressed cervical SC, as evidenced by a notable decrease in the total count of motoneuron soma ChAT and NeuN double-positive cells in the ventral horns within the compression zone, in a comparable fashion to previous reports (Karadimas et al., 2013). Additionally, following 12 weeks of SC compression, we observed an asymmetric loss of motoneurons between the ventral horns of the same region. This finding implies that intrinsic or extrinsic factors may contribute to the susceptibility or vulnerability of specific motoneurons. This may also imply that local environmental communication among resilient cells plays a pivotal role in the preservation of lower motoneurons. In this regard, ALS non-neuronal cells, including astrocytes, oligodendrocytes, microglial cells, and blood-derived immune cells, can contribute to the selective degeneration of motoneurons through the release of inflammatory mediators and cytokines, production of reactive oxygen species, clearance of neurotransmitters, neurotrophic support, and inorganic polyphosphates, among others (Crabé et al., 2020; Scamps et al., 2021; Arredondo et al., 2022). Changes in synaptic transmission and excitability of motoneurons are among the first events accompanying the deterioration of motoneuron circuitry in different motoneuron diseases (Mentis et al., 2011; Arumugam et al., 2017; Crabé et al., 2020; Scamps et al., 2021). Lower motoneurons receive inputs from local spinal networks, descending pathways, and sensory neurons (Mentis et al., 2011; Scamps et al., 2021), and, in particular, upper motoneurons are glutamatergic descending neurons that make synaptic contacts directly or indirectly via interneurons onto lower motoneurons through the corticobulbar and corticospinal tracts (Scamps et al., 2021). Our findings underscore that synaptic inputs to motoneurons undergo significant disruption 3 months after the DCM induction. Notably, our quantitative analysis of presynaptic boutons indicates that DCM is associated with synaptic remodeling originating from descending neurons, leading to a consequential reduction in positive glutamatergic synaptic inputs onto motoneuron somata. It is noteworthy that alterations in synaptic inputs to motoneurons, observed in conditions such as ALS and spinal muscular atrophy (SMA), can significantly impact various electrophysiological properties. For instance, changes may occur in action potential frequency and input resistance, potentially resulting in motoneuron hyperexcitability. These alterations are often associated with major defects in voltage-dependent sodium currents (Mentis et al., 2011; Liu et al., 2015; Arumugam et al., 2017; Scamps et al., 2021). DCM affects over 70% of the elderly population globally (Irvine et al., 1965; Moghaddamjou et al., 2020). Thus, it becomes imperative to deeply contemplate its implications for age-related motor deterioration. Within this framework, there is an ongoing debate regarding the fate of α-motoneurons and whether they undergo progressive degeneration, atrophy, or are subject to other underlying mechanisms. Prior empirical evidence does suggest a reduction in the number of motoneurons beyond the age of 60 years (Maxwell et al., 2018). However, contrasting studies propose that motoneurons are effectively maintained in cats and mice (Liu et al., 1996; Chai et al., 2011). Furthermore, research involving rhesus monkeys has intriguingly revealed that α-motoneurons not only retain both their size and number but also age at a differing pace (Maxwell et al., 2018), a feature probably related to the intrinsic cell properties and its differential vulnerability observed in pathological conditions such as ALS or SMA (Valdez et al., 2012; Comley et al., 2015; Murray et al., 2015; Tejero et al., 2016; Nijssen et al., 2017). In parallel, despite the maintenance of motoneurons, aging affects cholinergic and glutamatergic synaptic inputs of α-motoneurons (Maxwell et al., 2018), implying that DCM-associated aging could gradually exacerbate synaptic degeneration in the spinal cord.

Along with the alterations observed in the central nervous system, synaptic alterations in DCM extend to the peripheral NMJs at the level of SC compression. Our findings substantiate that skeletal muscle fibers are innervated by smaller NMJs upon DCM induction. Notably, there were no discernible structural defects indicative of denervation, axon sprouting beyond the postsynaptic apparatus, or postsynaptic fragmentation observed in the DCM mouse model. It is intriguing to note that certain myofibers exhibit poly-innervated synaptic sites, likely attributable to neuronal damage or potential compensatory response to motoneuron loss. These findings hold substantial importance in understanding motor deficits associated with DCM. Notably, our model deviates from traditional neuromuscular models characterized by partial or complete denervation, axonal swelling, and significant alterations in NMJ structure (Valdez et al., 2010; Torres-Benito et al., 2011; Bermedo-García et al., 2022). Partially, these disparities might arise from a more direct mechanism in which motoneurons and their corresponding target myofibers are concurrently lost, leaving the tissue with limited capacity to compensate for the damage. Supporting this notion at the cellular level, we observed a significant reduction in the size of the BB skeletal muscle fibers. Sarcopenia is a syndrome distinguished by the gradual decline of both skeletal muscle mass and strength (Cruz-Jentoft et al., 2010). This condition is associated with a reduction in the quantity of muscle fibers and a decrease in their cross-sectional area (Jun et al., 2023), a pattern that aligns with the evidence that we found in DCM mice. Also related to the characteristic muscle metabolism changes observed in sarcopenia, our results demonstrate an elevated ratio of oxidative type I muscle fibers, similar to what occurs during the natural aging process (Kirkendall and Garrett, 1998; Picard et al., 2011), even though this type of muscle fiber is more sensitive to inactivity and denervation-induced atrophy (Macpherson et al., 2011). Conversely, we did not observe any alterations in the glycolytic type IIB fibers despite prior reports highlighting the vulnerability of this group of skeletal muscle fibers to conditions such as cachexia and aging (Goodman et al., 2011; Wing et al., 2011; Fearon et al., 2012). Therefore, the enigmatic mechanisms that underlie the selective muscle fiber-type atrophy in DCM continue to elude our understanding, representing an intriguing open question for future research. Consistent with the muscle sarcopenia observed in DCM, our findings reveal a reduced number of central nuclei, which serves as an indicator of muscle regeneration. In this context, it reflects a pathological effect that potentially underscores a diminished capacity for muscle repair. Consequently, the deleterious consequences of DCM disrupt the maintenance of muscle mass, exerting a profound influence on muscular function and strength.

Currently, several studies link motor disorders with the homeostasis of gut microbiota and nutrient metabolism (Gungor et al., 2016; Zhang et al., 2018; Lin et al., 2020; Farini et al., 2023). From this standpoint, we explored the possibility that DCM mice could experience intestinal dysfunction as a neurogenic consequence, considering prior reports showing changes in gut microbiota composition after DCM (Farkas et al., 2023). In a mouse model of traumatic SCI, gut dysbiosis exacerbated the lesion size and neuroinflammation within the SC, inducing changes in gut-associated lymphoid cells and impairing neurological function. The locomotor disability in SCI mice was partially alleviated following a 5-week regime of probiotics enriched with lactic-producing bacteria administered post-injury (Kigerl et al., 2016). Consequently, cumulative efforts to foster connections between motoneurons and skeletal muscles now emphasize the potential of the gut microbiome both as a neuroprotective intervention and as a biomarker for monitoring ALS progression (Wu et al., 2015; Sun et al., 2019; Di Gioia et al., 2020; Obrenovich et al., 2020). Indeed, the SOD1^G93A^ transgenic mouse model of ALS exhibits increased gut permeability as a consequence of altered proteins conforming to tight junction structure, the first barrier of the intestinal epithelium (Wu et al., 2015), a phenomenon that is prevented by butyrate supplementation (Zhang et al., 2017). Consistently, treatments with butyrate restore intestinal homeostasis, prolonging the survival of SOD1^G93A^ mice (Zhang et al., 2017). Based on this evidence, we also studied the large intestinal barrier, resulting in an abundant and well-integrated epithelial barrier in DCM mice. Interestingly, the overall status of the colon was similar between the two groups. Nonetheless, we noted the presence of small mucin granules produced by goblet cells, indicating a degree of intestinal dysfunction. We also observed shifts in the abundance of phylum profiles, specifically in Firmicutes and Bacteroidetes, with an increased ratio between these two phyla. The imbalance in gut microbiota strongly implies alterations in microbiota-derived metabolites, notably marked by their association with a reduction in SCFAs, causing subsequent bowel inflammation (Stojanov et al., 2020). SCFAs are bacterial products derived from the fermentation of dietary fiber in the gastrointestinal tract. Along with the colon, the most abundant SCFAs in the human body are acetate, propionate, and butyrate, in the approximate molar ratio of 60:20:20, respectively (Macfarlane and Macfarlane, 2003). The SCFAs contribute to the maintenance of gut health. Locally, butyrate and acetate, but not propionate, increase mucus production in the colon (Barcelo et al., 2000), suggesting that propionate may influence other tissues. In fact, SCFAs display multiple direct or indirect roles in the central nervous system (CNS), including the regulation of epigenetics, neuroinflammation, neuroendocrine responses, brain metabolism, and protein synthesis, among other processes (Dalile et al., 2019). To evaluate this possibility, we challenged the motoneuron-like NSC34 cell line with acetate, butyrate, and propionate and found remarkable effects in the length of processes, suggesting that gut microbiota metabolites could influence the maintenance of motoneurons. Collectively, these findings make evident that targeting the gut microbiota holds promise as a valuable approach to ameliorate spinal cord disabilities, including DCM, through the restoration of intestinal homeostasis and the equilibrium of its metabolites. For instance, a direct temporal correlation of NMJ detriment with dysbiosis, immunophenotypes, and epigenetics marks was found in the ALS mouse model (Figueroa-Romero et al., 2020). Interestingly, changes in microbiome precede the onset of locomotor deficits (muscle atrophy, grip strength, and motor coordination) in transgenic SOD1^G93A^ mice (Figueroa-Romero et al., 2020). Therefore, in our model, it is conceivable that intestinal dysfunction may manifest after 3 months of DCM.

Our study has genuine limitations. For instance, without the intention of perpetuating a historical gender bias, we use only male mice to evaluate all the parameters presented here. Future studies should compare females and males given the contribution of sex-related biological factors and the compensatory phenotypes observed in some neuromuscular diseases, especially energy metabolism and contractile properties of muscles (Glenmark et al., 2004; Deenen et al., 2015; Deschenes et al., 2018; Farkas et al., 2023; Landen et al., 2023; Levy et al., 2023; Vinciguerra et al., 2023). On the other hand, human and murine gut microbiota share over 80–90% similarities in phyla and genera, suggesting a high gut microbiota resembling. However, a detailed analysis has revealed discrepancies in the microbial abundance, including the ratio between Firmicutes to Bacteroidetes, as well as the presence of exclusive genera between humans and rodents (Guinane et al., 2013; Krych et al., 2013; Alkadhi et al., 2014; Nagpal et al., 2018). Finally, a multidirectional communication network for the maintenance of gastrointestinal homeostasis includes the integration of different systems (i.e., the central nervous system, the autonomic nervous system, the enteric nervous system, and the endocrine system). Thus, all these lines of communication must be dissected to allow a better comprehension of the basic mechanisms affected during DCM progression and development. Therefore, longitudinal studies integrating the communication between all these systems are necessary.

Based on our current findings and previous evidence, we postulate that DCM could be visualized as a synaptopathy, as it disrupts cellular communication crucial for motor function, thereby precipitating the decline of the entire neuromotor axis, ultimately culminating in muscle atrophy. Thus, our findings shed light on the development of fatigue, one of the symptoms reported by DCM patients whose etiology is uncertain. Furthermore, DCM may exert its influence on the dysfunction of other systems, particularly with a pronounced impact on the colon, leading to significant alterations in both the mucosal barrier and the delicate equilibrium of the intestinal microbiota.

## Data availability statement

The original contributions presented in the study are publicly available. This data can be found at: https://www.ncbi.nlm.nih.gov/bioproject/917190.

## Ethics statement

Ethical approval was not required for the studies on humans in accordance with the local legislation and institutional requirements because only commercially available established cell lines were used. The animal study was approved by Animal Use Committee of the Universidad Católica de la Santísima Concepción. The study was conducted in accordance with the local legislation and institutional requirements.

## Author contributions

JO: Conceptualization, Data curation, Formal analysis, Funding acquisition, Investigation, Methodology, Writing – original draft, Writing – review & editing. MV: Data curation, Formal analysis, Investigation, Writing – original draft. AÁ: Investigation, Writing – original draft, Writing – review & editing. JH: Investigation, Writing – review & editing. MF: Investigation, Resources, Writing – review & editing. PV: Conceptualization, Data curation, Funding acquisition, Investigation, Methodology, Supervision, Writing – original draft, Writing – review & editing.
