# Supplementary material for: Impaired communication at the neuromotor axis during Degenerative Cervical Myelopathy
Source: Front Cell Neurosci. 2024 Jan 10;17:1316432. doi: 10.3389/fncel.2023.1316432 (PMC10806149; doi:10.3389/fncel.2023.1316432)
Supplement: Supplementary file 1 [file Data_Sheet_1.docx]

Supplementary Material

## Supplementary Figures


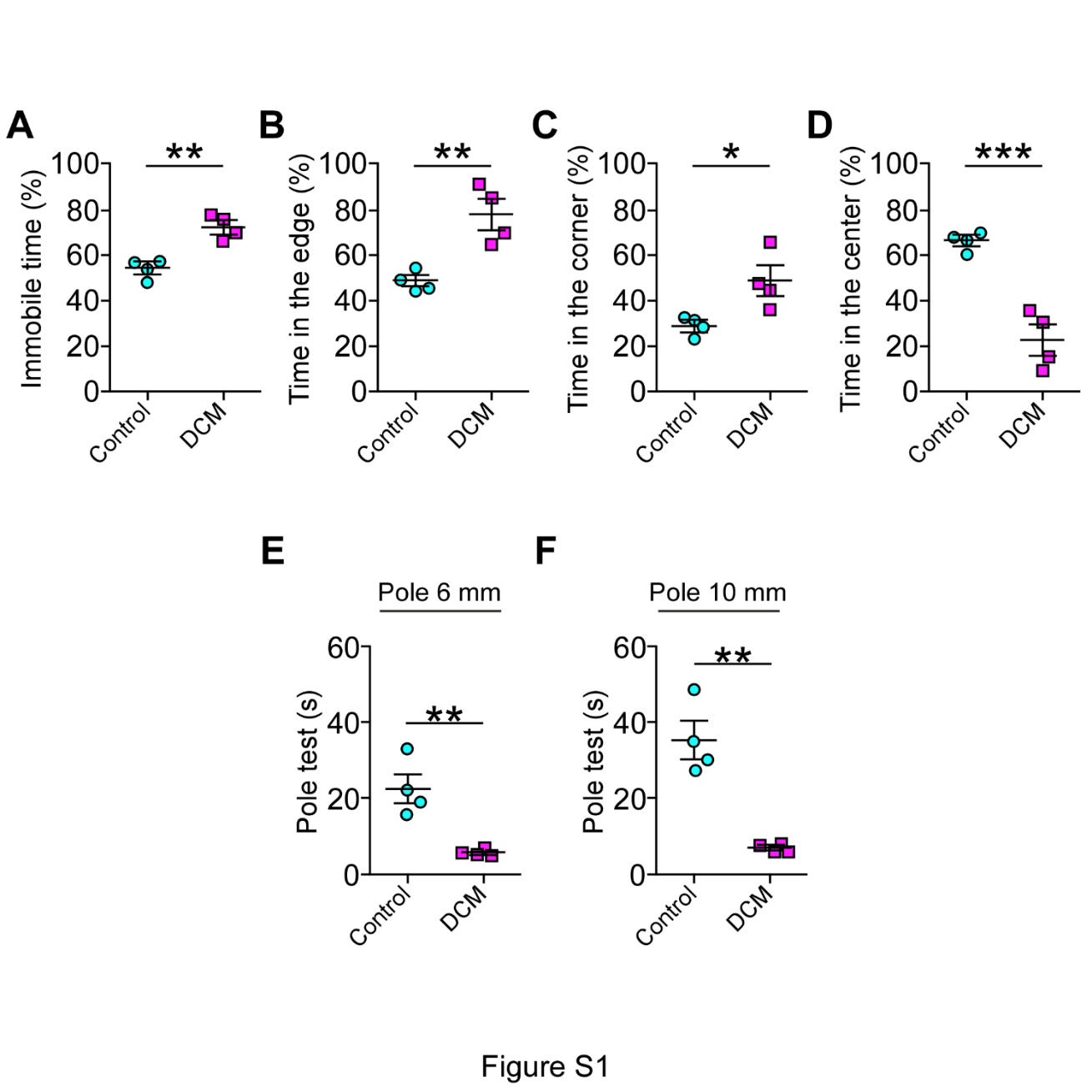


**Supplementary Figure 1.** **Mice present engaged motor performance after DCM.** In the open field test**,** the group of DCM mice spent more time (**S1A**) immobile (control: 54.1 ± 2.4 %; DCM: 71.9 ± 3.0 %; **p < 0.0039 *t-test*), specially at the (**S1B**) edges (control: 48.7 ± 2.2 %; DCM: 77.7 ± 6.8 %; **p < 0.0063 *t-test*) and on the (**S1C**) corners (control: 28.5 ± 2.5 %; DCM: 48.5 ± 6.4 %; *p < 0.028 *t-test*) than the (**S1D**) center (control: 66 ± 2.4 %; DCM: 22.2 ± 6.6 %; ***p < 0.0008 *t-test*) of the box. Cognitive damage on DCM mice was discard by using the pole test at **(S1E)** 6 mm (control: 20.2 ± 4.8 s; DCM: 6.7 ± 1.6 s; **p < 0.021 *t-test*) and **(S1F)** 10 mm (control: 35.2 ± 4.8 s; DCM: 7.00 ± 0.5 s; **p < 0.0012 *t-test*) of the bar diameter. Data are presented as mean ± s.e.m.; n= 4 per group.


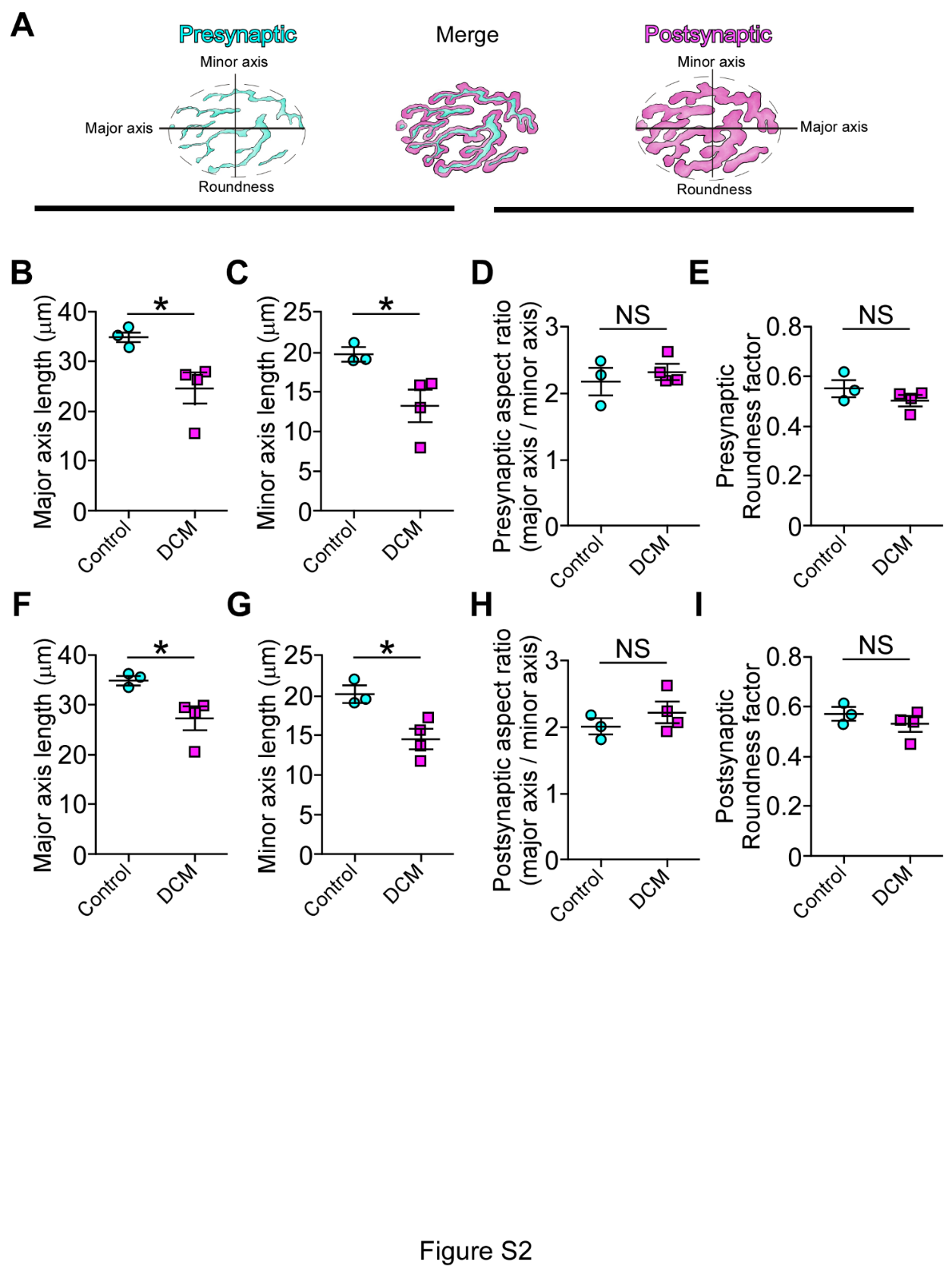


**Supplementary Figure 2.** **The DCM impairs the neuromuscular junction**. (**S2A**) The morphometric analysis of 200 µm thick slices from supraespinatus muscles were stained with neurofilament/SV2 (cyan) antibodies and αBTX (magenta) (figure 4A). The quantification of (**S2B**) major axis (control: 34.9 ± 0.8 µm; DCM: 24.5 ± 3.1 µm; *p < 0.048 *t-test*), (**S2C**) minor axis (control: 19.4 ± 0.8 µm; DCM: 13.1 ± 1.9 µm; *p < 0.045 *t-test*), (**S2D**) aspect ratio (control: 2.1 ± 0.2; DCM: 2.3 ± 0.1; p < 0.52 *t-test*) and (**S2E**) roundness factor (control: 0.5 ± 0.03; DCM: 0.50 ± 0.02; p < 0.24 *t-test*) for the presynaptic axon terminal and the (**S2F**) major axis (control: 34.9 ± 0.81 µm; DCM: 27.3 ± 2.3 µm; *p < 0.0442 *t-test*), (**S2G**) minor axis (control: 20.0 ± 1.0 µm; DCM: 14.4 ± 1.2 µm; *p < 0.0201 *t-test*), (**S2H**) aspect ratio (control: 2.0 ± 0.1; DCM: 2.2 ± 0.1; p < 0.0369 *t-test*) and (**S2I**) roundness factor (control: 0.5 ± 0.02; DCM: 0.5 ± 0.03; p < 0.3911 *t-test*) for the postsynaptic endplate support a neuromuscular impairment in DCM group compared with the control. Data are presented as mean ± s.e.m.; n control = 3, n DCM =4.


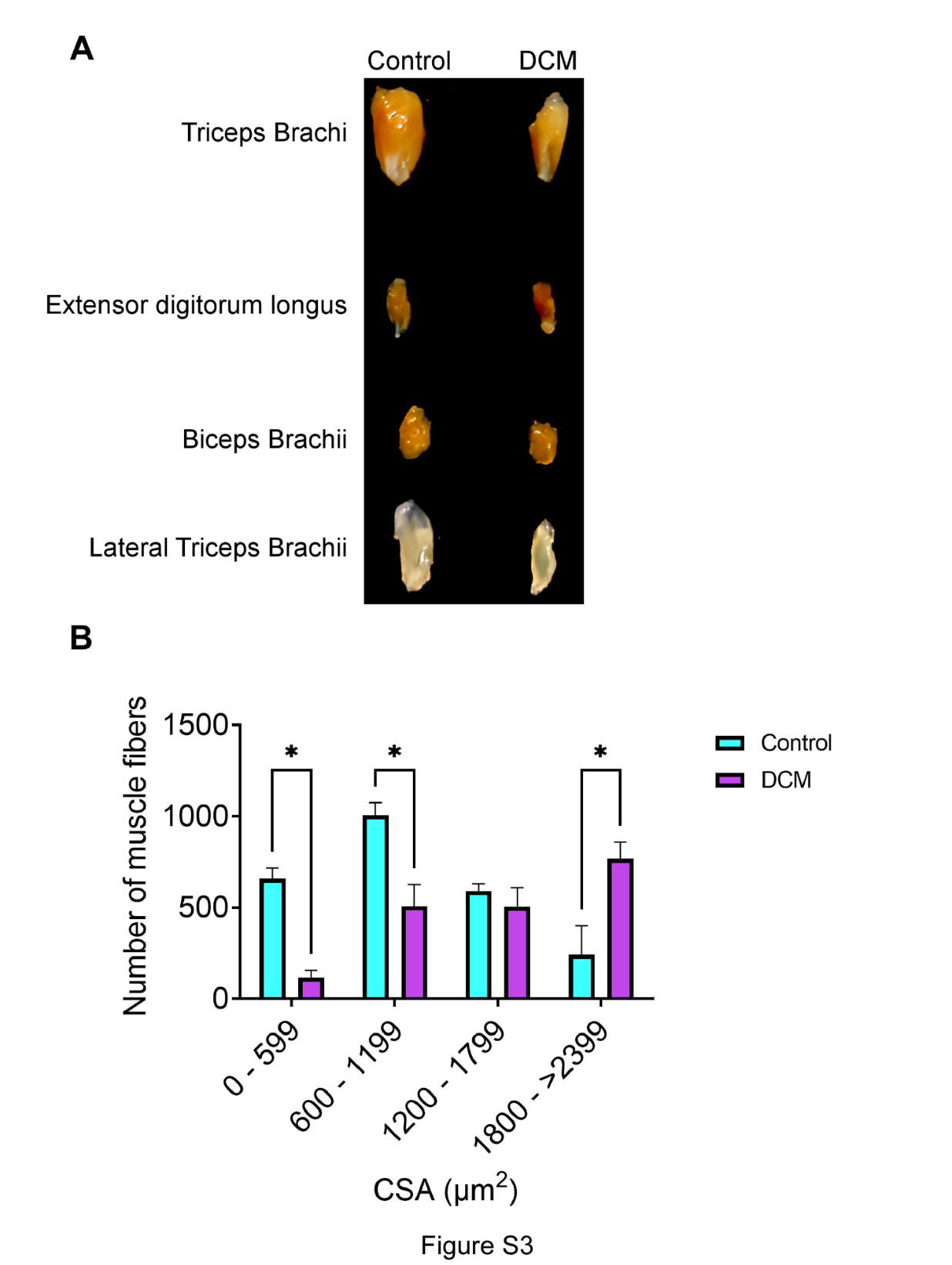


**Supplementary Figure 3.** Supplementary Figure S3. DCM impairs skeletal muscle tissue. (S3A) Whole muscles innervated by C5-6 spinal cord section were examined in control and DCM observing severe muscle wasting. (S3B) Distribution histogram of the cross-sectional area of biceps brachii in Control and DCM groups (control - DCM, 0-599: 656 ± 105 – 116 ± 68; 600-1199: 1005 ± 120 – 506 ± 209; 1200-1799: 590 ± 70 – 506 ± 178; 1800-2399: 240 ± 279 – 766 ± 160; *p < 0.016). The results represent the mean ± s.e.m. of n: 3 mice per group.
